# Supplementary material for: PAK1, PAK1Δ15, and PAK2: similarities, differences and mutual interactions
Source: Sci Rep. 2019 Nov 20;9:17171. doi: 10.1038/s41598-019-53665-6 (PMC6868145; doi:10.1038/s41598-019-53665-6)

PAK1, PAK1Δ15, and PAK2: similarities, differences, and mutual interactions

Dana Grebeňová, Aleš Holoubek, Pavla Röselová, Adam Obr, Barbora Brodská, Kateřina Kuželová

Supplementary Figures

Figure S1:**Comparison of signals from different PAK antibodies**. HEK293T cell lysate was resolved on a large gel, the proteins were transfered to a nitrocellulose membrane. The membrane was vertically cut, the individual parts were incubated with the indicated primary antibody, then with the corresponding secondary antibody. The membrane was reassembled, covered with the chemiluminiscence substrate and the signal was recorded from all the parts at once. Detailed description of antibodies used is given in Table 1 (main text).

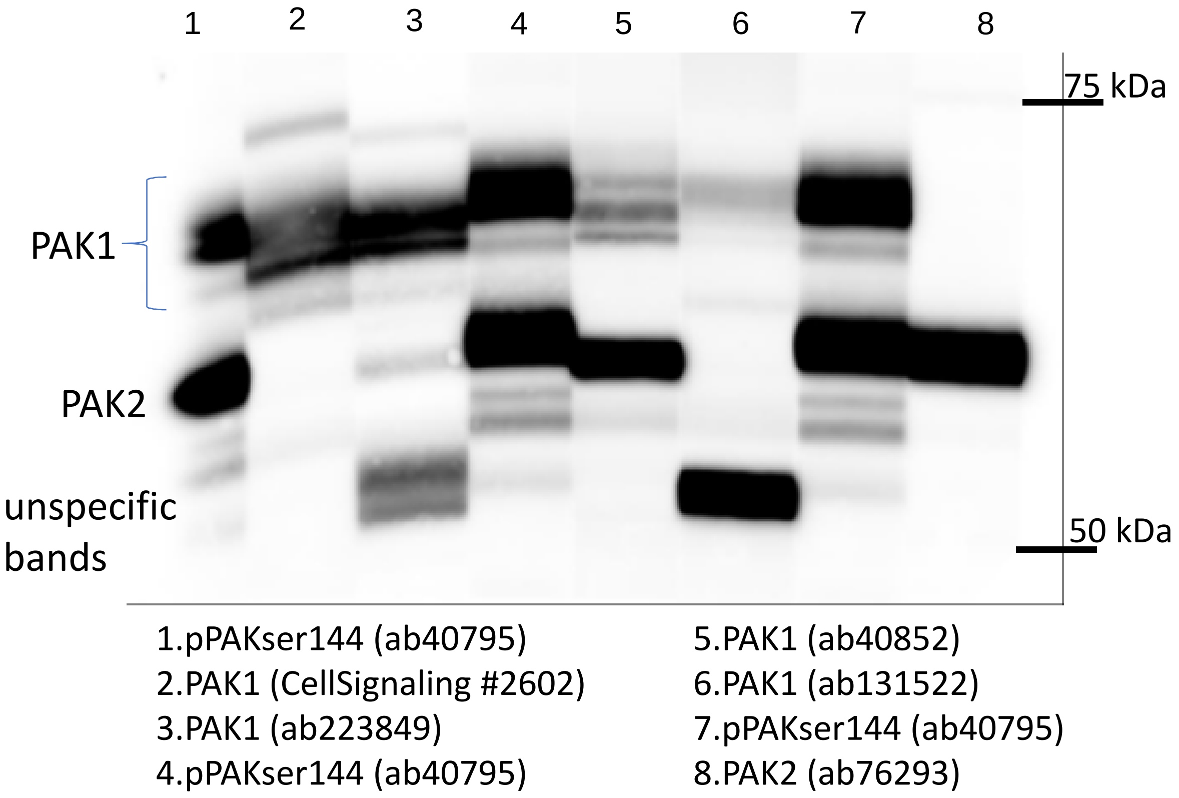

Figure S2: **Sequence comparison of PAK isoforms**
Triple sequence alignment was performed using Clustal W software.

```
                        1-50
PAK1 full length        MSNNGLDIQDKPPAPPMRNTSTMIGAGSKDAGTLNHGSKPLPPNPEEKKK
PAK1 delta15            MSNNGLDIQDKPPAPPMRNTSTMIGAGSKDAGTLNHGSKPLPPNPEEKKK
PAK2                    MSDNG-ELEDKPPAPPVRMSSTIFSTGGKDPLSANHSLKPLPSVPEEKKP
                        51-100
PAK1 full length        KDRFYRSILPGDKTNKKKEKERPEISLPSDFEHTIHVGFDAVTGEFTGMP
PAK1 delta15            KDRFYRSILPGDKTNKKKEKERPEISLPSDFEHTIHVGFDAVTGEFTGMP
PAK2                    RHKIISIFSGTEKGSKKKEKERPEISPPSDFEHTIHVGFDAVTGEFTGMP
                        101-150
PAK1 full length        EQWARLLQTSNITKSEQKKNPQAVLDVLEFYNSKKTSNSQKYMSFTDKSA
PAK1 delta15            EQWARLLQTSNITKSEQKKNPQAVLDVLEFYNSKKTSNSQKYMSFTDKSA
PAK2                    EQWARLLQTSNITKLEQKKNPQAVLDVLKFYDSNT--VKQKYLSFTPPEK
                        151-199
PAK1 full length        EDYNS-SNALNVKAVSETPAVPPVSEDEDDDDDDATPPPVIAPRPEHTKS
PAK1 delta15            EDYNS-SNALNVKAVSETPAVPPVSEDEDDDDDDATPPPVIAPRPEHTKS
PAK2                    DGFPSGTPALNAKG-TEAPAV--VTEEEDD--DEETAPPVIAPRPDHTKS
                        200-249
PAK1 full length        VYTRSVIEPLPVTPTRDVATSPISPTENNTTPPDALTRNTEKQKKKPKMS
PAK1 delta15            VYTRSVIEPLPVTPTRDVATSPISPTENNTTPPDALTRNTEKQKKKPKMS
PAK2                    IYTRSVIDPVPA-PVGD-------------SHVDGAAKSLDKQKKKTKMT
                        250-299
PAK1 full length        DEEILEKLRSIVSVGDPKKKYTRFEKIGQGASGTVYTAMDVATGQEVAIK
PAK1 delta15            DEEILEKLRSIVSVGDPKKKYTRFEKIGQGASGTVYTAMDVATGQEVAIK
PAK2                    DEEIMEKLRTIVSIGDPKKKYTRYEKIGQGASGTVFTATDVALGQEVAIK
                        300-349
PAK1 full length        QMNLQQQPKKELIINEILVMRENKNPNIVNYLDSYLVGDELWVVMEYLAG
PAK1 delta15            QMNLQQQPKKELIINEILVMRENKNPNIVNYLDSYLVGDELWVVMEYLAG
PAK2                    QINLQKQPKKELIINEILVMKELKNPNIVNFLDSYLVGDELFVVMEYLAG
                        350-399
PAK1 full length        GSLTDVVTETCMDEGQIAAVCRECLQALEFLHSNQVIHRDIKSDNILLGM
PAK1 delta15            GSLTDVVTETCMDEGQIAAVCRECLQALEFLHSNQVIHRDIKSDNILLGM
PAK2                    GSLTDVVTETCMDEAQIAAVCRECLQALEFLHANQVIHRDIKSDNVLLGM
                        400-449
PAK1 full length        DGSVKLTDFGFCAQITPEQSKRSTMVGTPYWMAPEVVTRKAYGPKVDIWS
PAK1 delta15            DGSVKLTDFGFCAQITPEQSKRSTMVGTPYWMAPEVVTRKAYGPKVDIWS
PAK2                    EGSVKLTDFGFCAQITPEQSKRSTMVGTPYWMAPEVVTRKAYGPKVDIWS
                        450-499
PAK1 full length        LGIMAIEMIEGEPPYLNENPLRALYLIATNGTPELQNPEKLSAIFRDFLN
PAK1 delta15            LGIMAIEMIEGEPPYLNENPLRALYLIATNGTPELQNPEKLSAIFRDFLN
PAK2                    LGIMAIEMVEGEPPYLNENPLRALYLIATNGTPELQNPEKLSPIFRDFLN
                        500-548
PAK1 full length        RCLEMDVEKRGSAKELLQVRKLRFQ-VFSNFSMIAASIPEDCQAPLQPHS
PAK1 delta15            RCLEMDVEKRGSAKELLQHQFLKIAKPLSSLTPLIAAAK---------EA
PAK2                    RCLEMDVEKRGSAKELLQHPFLKLAKPLSSLTPLIMAAK---------EA
                        549-553
PAK1 full length        TDCCS
PAK1 delta15            TKNNH
PAK2                    MKSNR
```

Figure S3: **Assessment of PAK transcript levels in HEK293T cells.**
Total RNA was extracted from HEK293T cells using Rneasy Mini Kit (Qiagen) and cDNA was synthesized with SensiFast cDNA Synthesis Kit (Bioline). Individual mRNA expression levels were evaluated from cDNA by PCR amplification (SensiFast SYBR No-ROX Kit, Bioline) using primers for PAK1 full-length (Fw:AGAGCTGCTACAGGTGAGAA, Rv:GCAATCAGTGGAGTGAGGCT), PAK1Δ15 (Fw:GAGCTGCTACAGCATCAATTCC, Rv:GCAAGGAGAAGAGGGCATCA), PAK2 (Fw:CCCTTGAGGGCCTTGTACCT, Rv:CGGTTTGGCCAGTTTCAGGA), and GAPDH (Fw:GAAACTGTGGCGTGATGGC, Rv:CCGTTCAGCTCAGGGATGAC) .
The graph shows the ratio of PAK1-full, resp. PAK1Δ15, to PAK2.

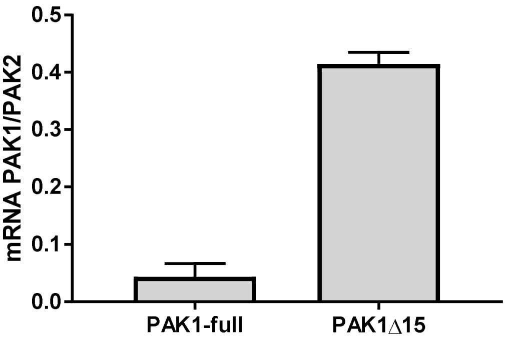

Figure S4: **Detection of the truncated PAK2-eGFP in immunoprecipitates**
HEK293T cells were transfected with PAK2-eGFP in combination with PAK1-full-mCherry or with a control mCherry plasmid. The immunoprecipitates obtained from beads binding mCherry/RFP or GFP were probed by a GFP antibody. The truncated PAK2 was not found in the absence of PAK1-full (lane 3).

1. PAK2-eGFP + PAK1-full-mCherry, IP:RFP
2. PAK2-eGFP + PAK1-full-mCherry, IP:GFP
3. PAK2-eGFP + empty mCherry, IP:GFP

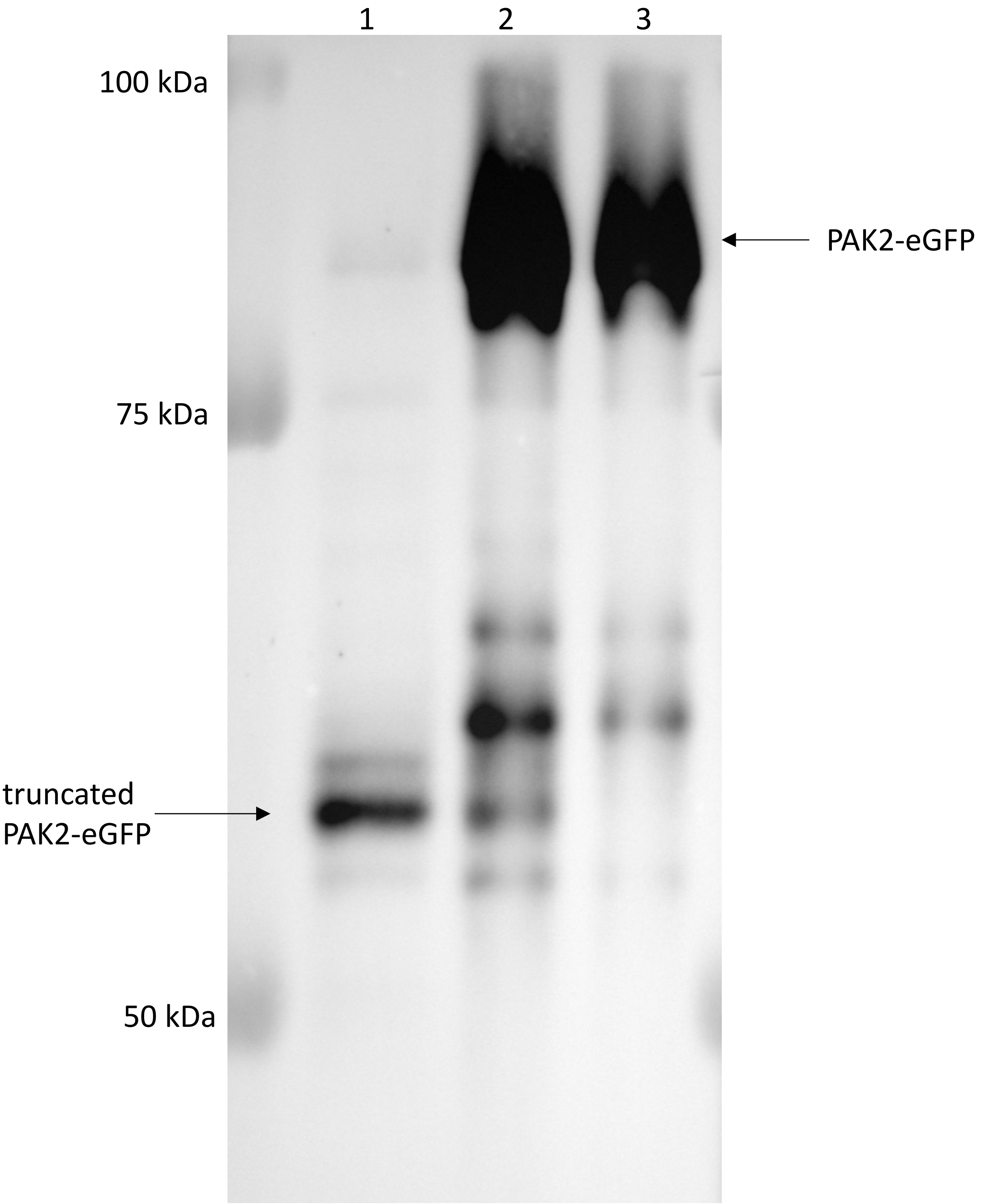

Figure S5a: **Co-immunoprecipitation with inverse labeling**
HEK293T cells were cotransfected with combinations of labeled PAK isoforms (G-green variant, R-red variant) and empty eGFP/mCherry plasmids. Proteins were precipitated through GFP or RFP beads (indicated as IP:GFP or IP:RFP). The membranes were probed with anti-GFP or anti-RFP antibodies (indicated as WB:GFP or WB:RFP). The input (lysate) is also shown in the left column. MW markers are shown on the left of each blot.

1 PAK1-full-eGFP + PAK2-mCherry
2 PAK1-full-eGFP + mCherry

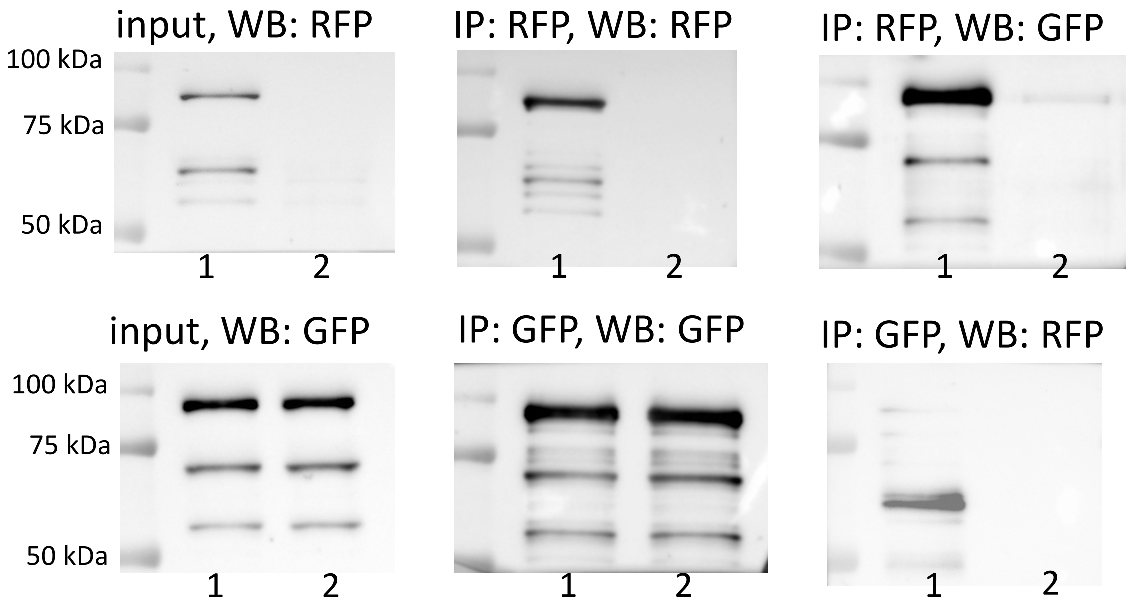

Figure S5b: **Effect of caspase inhibition on PAK2-eGFP truncation**
HEK293T cells were transfected with the indicated plasmids in the absence or in the presence of 10 µM Q-VD-OPh (added 1 h prior to transfection and maintained until the cell harvest). PAK were precipitated using GFP/RFP beads and the presence of interacting partners was analyzed using RFP/GFP antibody.

1 PAK2-eGFP + PAK1-full-mCherry
2 PAK2-eGFP + PAK1-full-mCherry + Q-VD-OPh
3 PAK2-eGFP + empty mCherry
4 PAK2-eGFP + empty mCherry + Q-VD-OPh

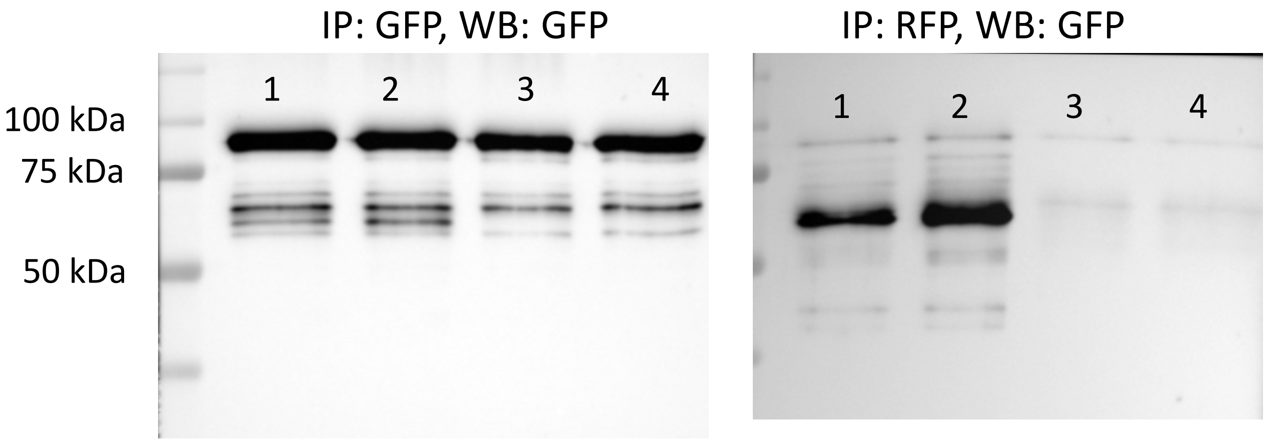

The efficiency of Q-VD-OPh was verified through analysis of PARP cleavage:

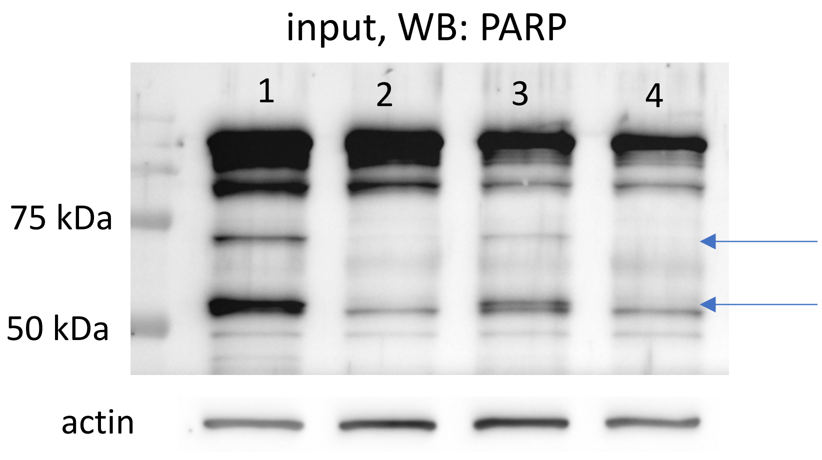

Figure S6: (a) **Detection of the endogenous PAK1 in immunoprecipitates.** HEK293T cells were transfected with the plasmid encoding PAK1-full-GFP. The exogenous PAK1 with its interaction partners was pulled down using GFP-trap beads. The precipitate was resolved using SDS electrophoresis and blotted to two membranes, which were incubated with antibodies against GFP (left) or PAK1 (ab223849, right). The position of MW markers is indicated on the left side. The blue arrow points to the expected position of the endogenous PAK1 (64-67 kDa). No band was detected at this position using the GFP antibody.

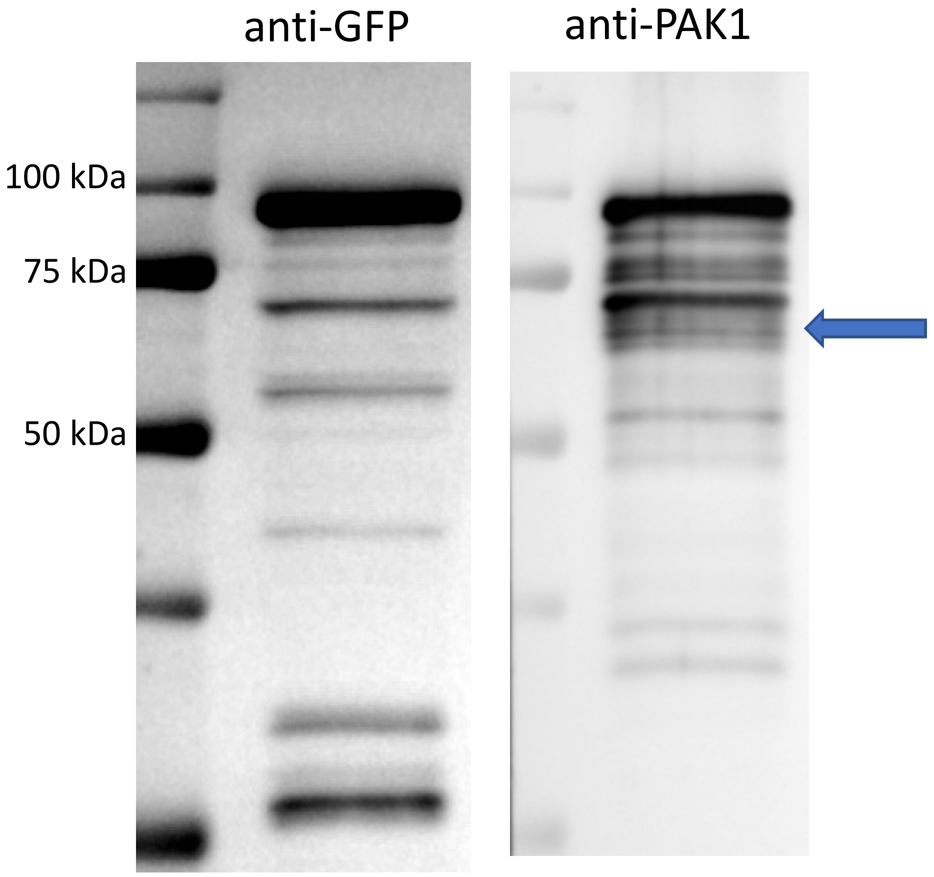

(b) **Ser144/141 PAK phosphorylation in cells transfected with PAK-eGFP.** HEK293T cells were transfected with the plasmids encoding PAK1-full-eGFP, PAK1Δ15-eGFP or PAK2-eGFP and the phosphorylation level was assessed after 24h.

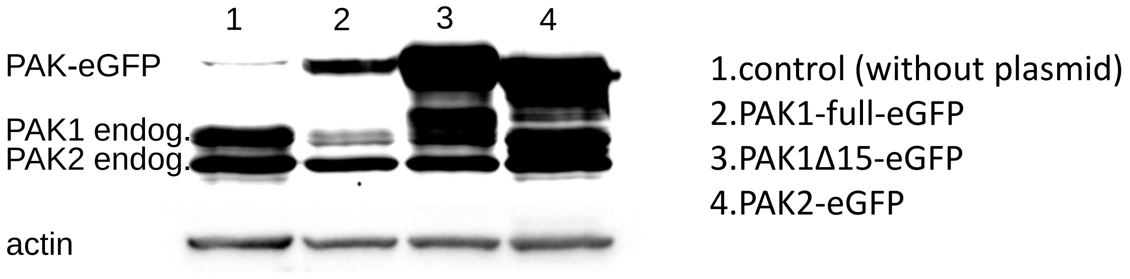

Figure S7: **PAK intracellular localization.** Additional examples of confocal microscopy images of single transfected (a,b) or co-transfected (c) HeLa cells. This Figure complements Fig. 7 of the main text.

(a) PAK2-eGFP localization in focal adhesions, which are stained by paxillin or vinculin in fixed HeLa cells

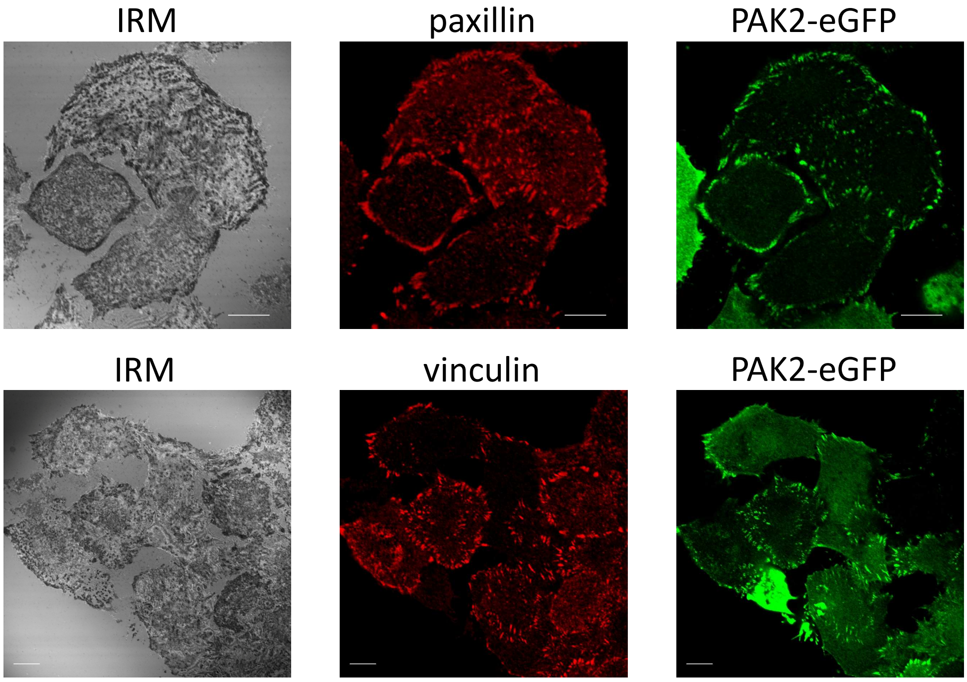

(b) PAK1Δ15-eGFP localization in focal adhesions, which are visualized by interference reflection microscopy (IRM) in living cells or by paxillin staining in fixed cells

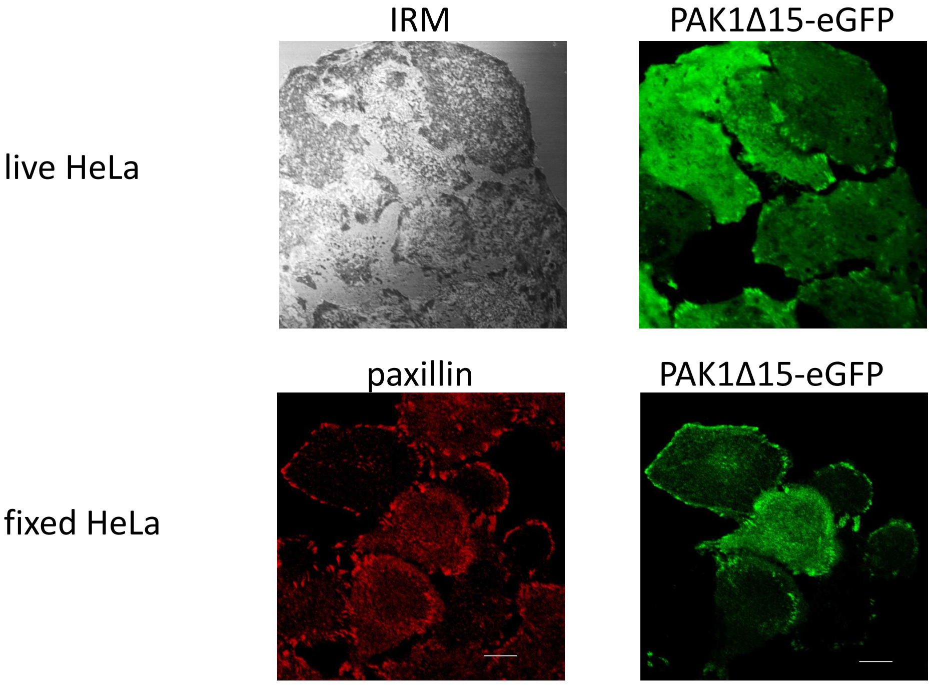

Figure S7 - suite

(c) comparison of PAK1-full (red) localization with that of PAK2/PAK1Δ15 (green) in live cotransfected HeLa cells

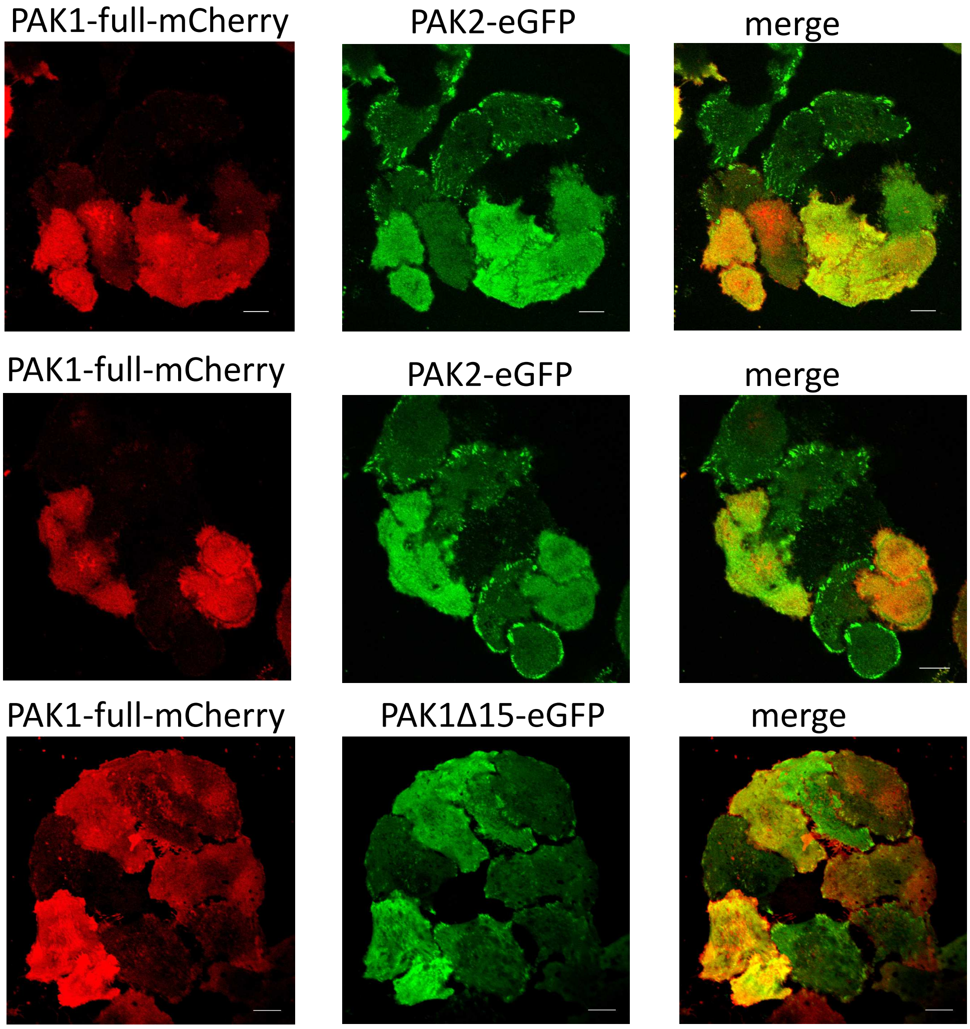

Figure S8 **Comparison of PAK1 and PAK2 expression level in HeLa and HEK293T cells.** Lysates from HEK293T and HeLa cells were resolved in 18x18 cm gels, the proteins were transferred to a membrane and visualized using the indicated antibodies.

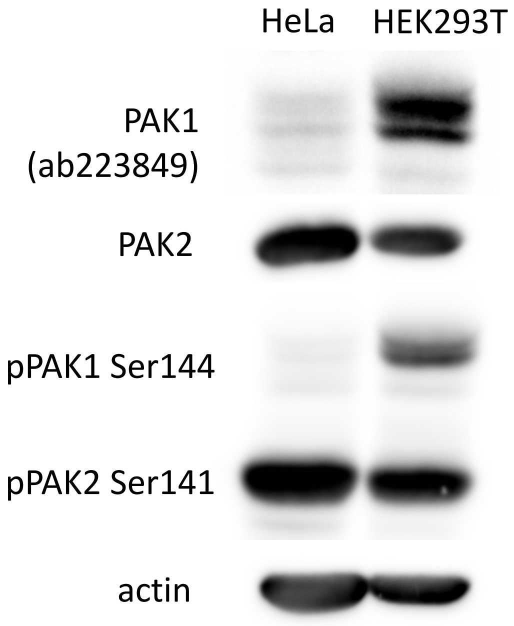

Figure S9:Analysis of endogenous PAK localization by immunofluorescence staining. Microtubule organizing centers stained by the phospho-specific (Ser144/141) antibody to PAK1/PAK2 in mitotic cells. Green – pPAK1+pPAK2, blue – nuclei. Scale bars: 10 µm.

HEK293T

HeLa

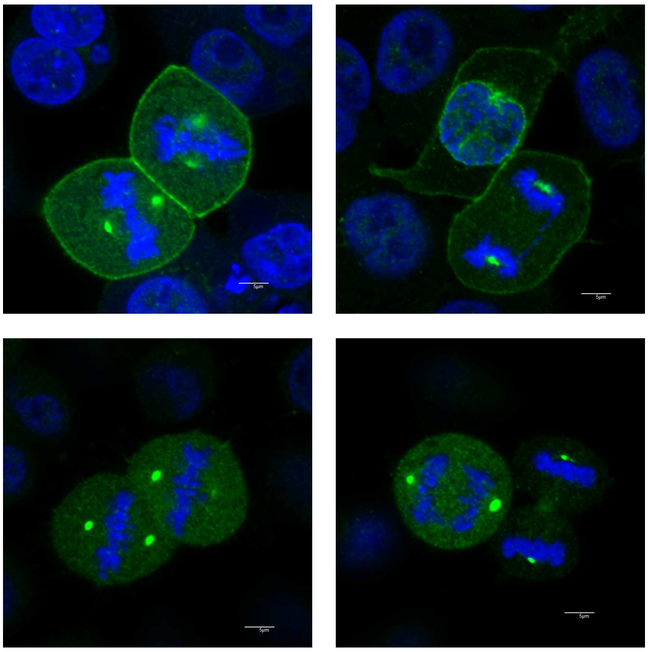

Figure S10: **Cell viability after 24 h treatment with IPA-3 and PIR3.5**
HeLa cells were harvested from plates at the end of ECIS measurement, 24 h after inhibitor addition (setting 2). Cell viability was measured by the standard propidium iodide (PI) exclusion assay using the flow-cytometer BD Fortessa. Means and s.d. from 5 to 9 experiments for each condition.

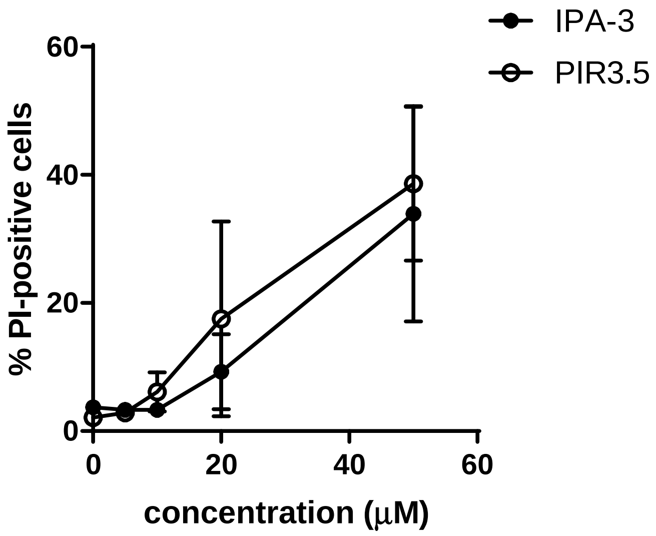

Figure S11: **IPA-3 or PIR3.5-induced changes in PAK Ser144/141 phosphorylation**
The cells were treated for 30 min with different concentrations of IPA-3 or PIR3.5. Means and s.d. from at least 3 repeated experiments for each condition. The individual phospho-PAK bands are defined in Figure 1b (main text).

a. HEK293T cells treated with PIR3.5 in suspension or as an adhered monolayer

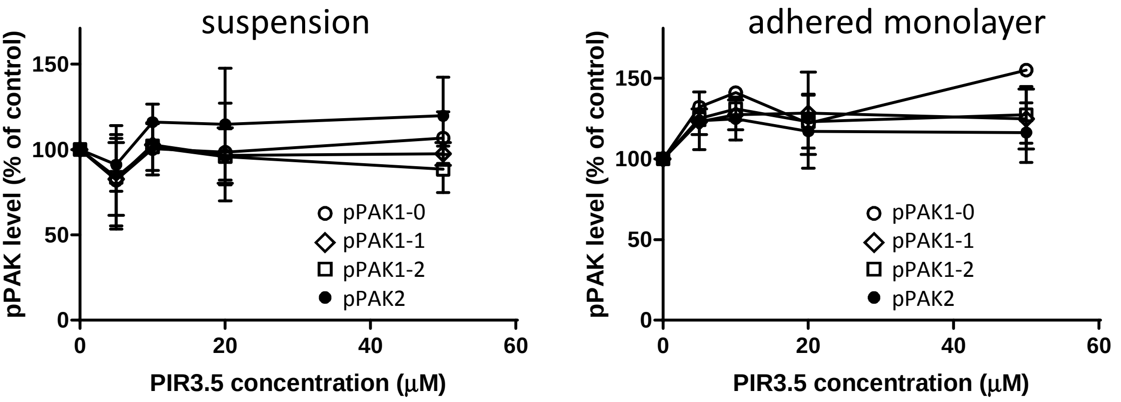

b. HeLa cells treated with IPA-3 (upper plots) or PIR3.5 (lower plots) in suspension or as an adhered monolayer. The symbols are the same as for the panel a.

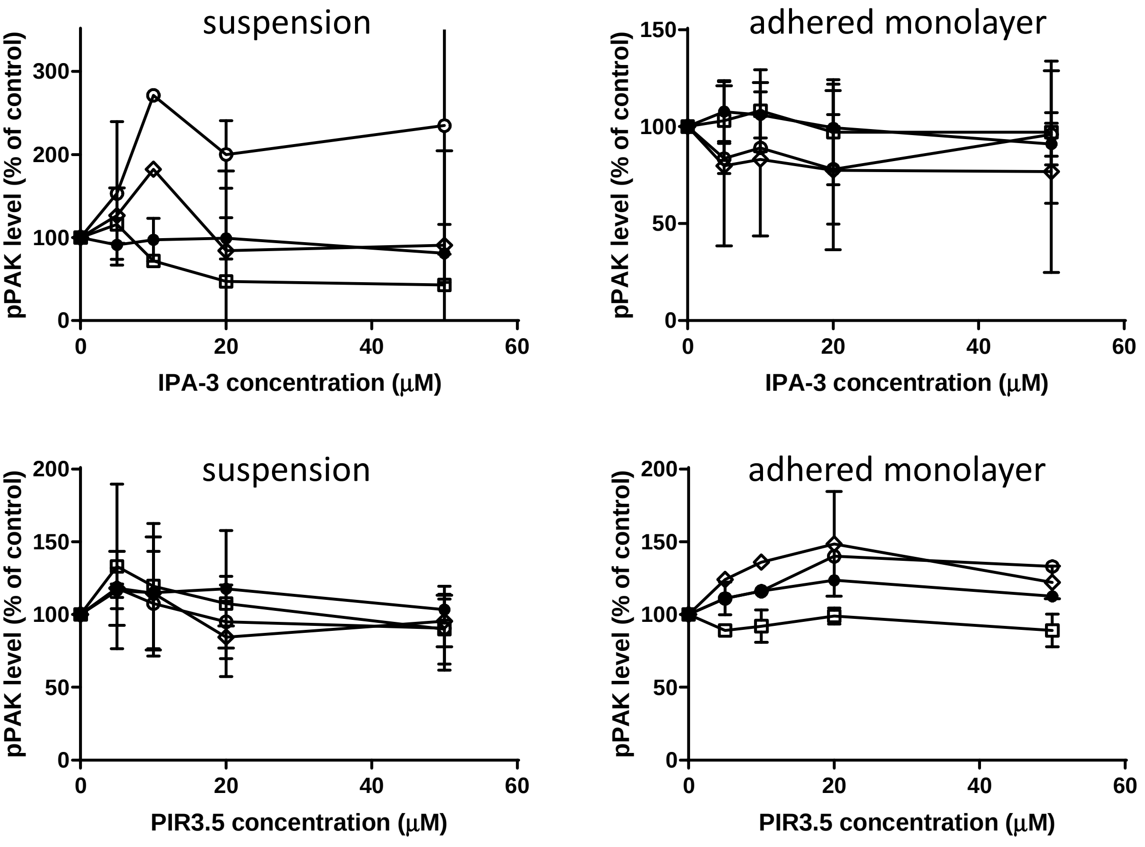

Figure S12: **IPA-3-induced changes in T212 and Ser20 phosphorylation**
HEK293T cells in suspension were treated for 30 min with IPA-3. The lysates were resolved by gel electrophoresis and the western-blot membranes were incubated with phospho-specific antibodies recognizing pT212 (ab75599) or pSer20 (ab51244) of PAK1. The figure shows examples of the signals (upper part) and the summary of the results obtained (means and ranges from 7 and 5 experiments for T212 and Ser20, respectively).

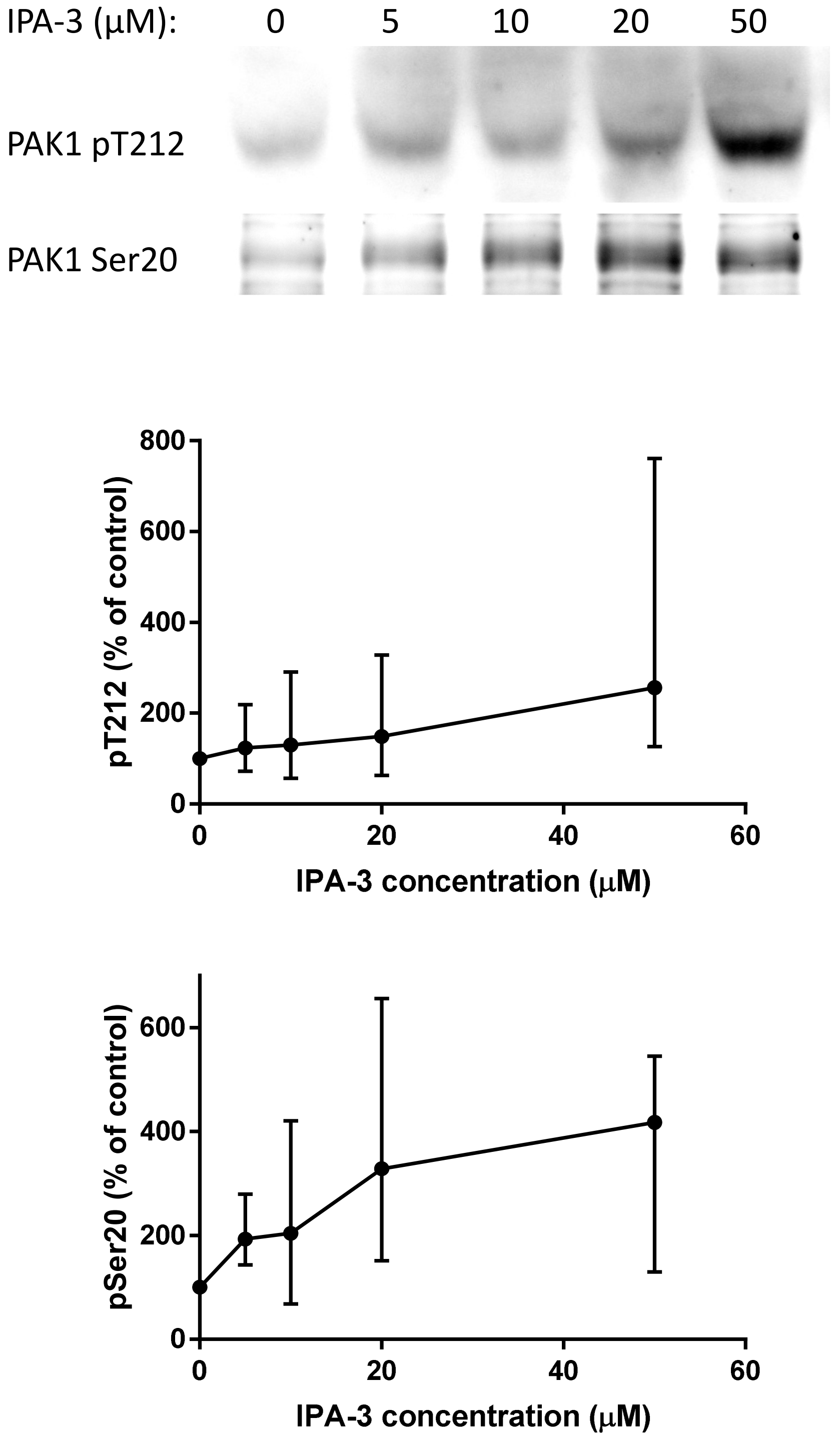

Figure S13: **Morphological changes induced by dasatinib**
Cells were treated for 90 min with 100 nM dasatinib and their morphology was examined using the Nanolive microscope (Agilent). A: HEK293T cells, representative examples of non-treated and treated cells. B: HeLa cells. The plot documents a significant decrease in the mean cell surface area after dasatinib treatment.

A. HEK293T

control

dasat

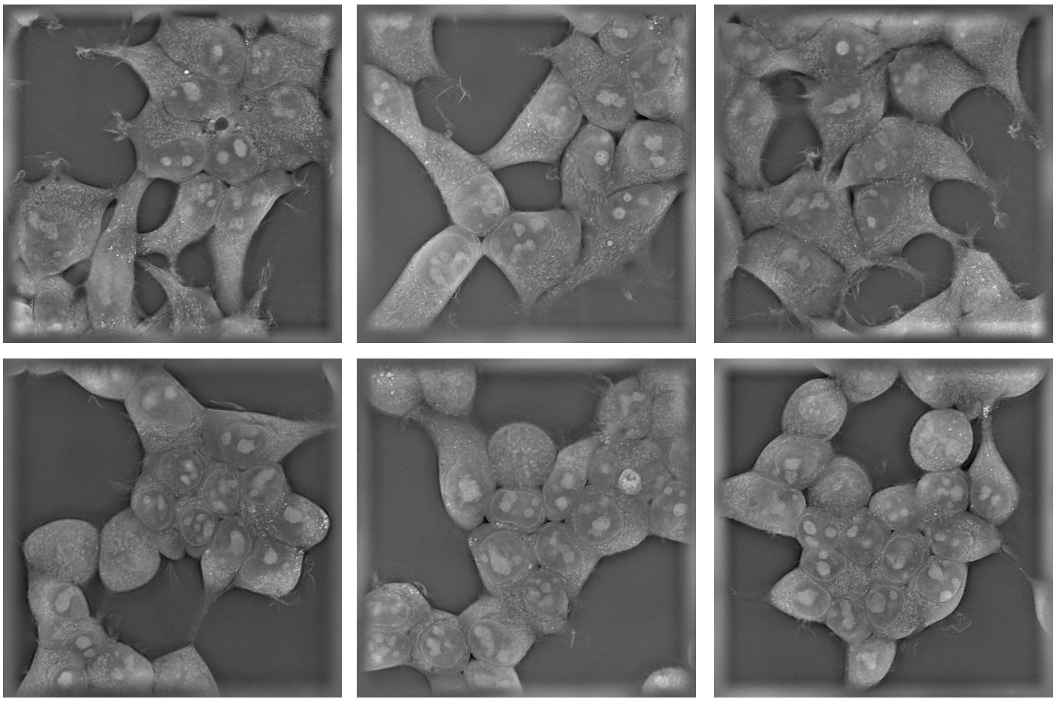

B. HeLa

control

dasat

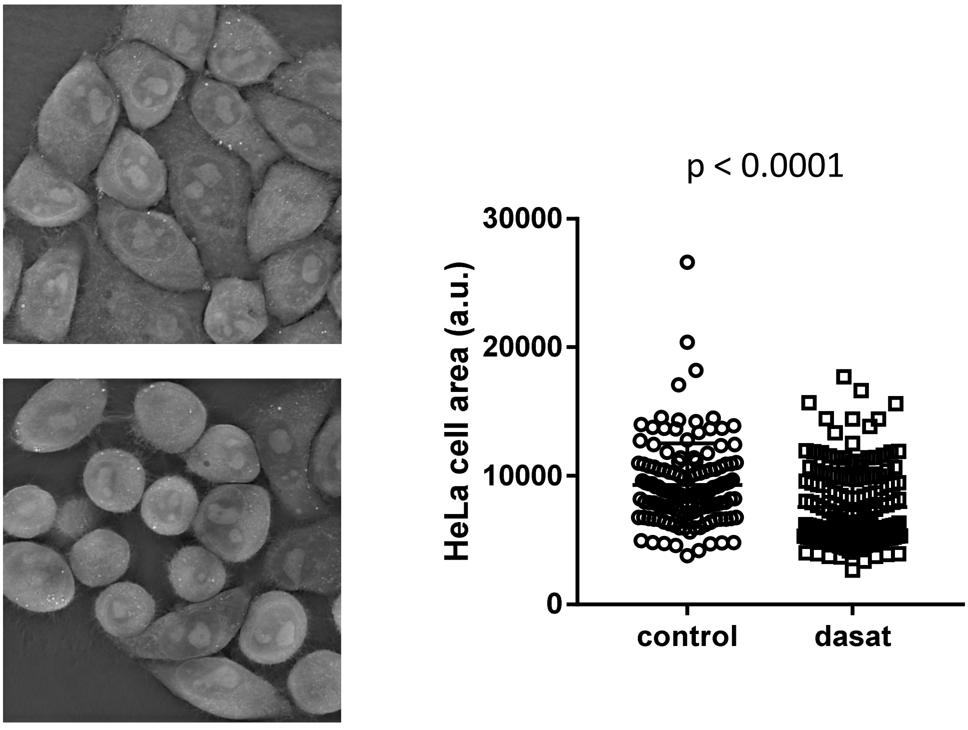

Figure S14: **Representative examples of ECIS records for PIR3.5 treatment**
HeLa cells (upper plots) or HEK293T cells (lower plots) were preatreated for 30 min with inhibitors before seeding to ECIS wells (left) or treated during measurement (right). The arrows mark the time of inhibitor addition. Color legend: controls: black, PIR3.5 at 5 – 10 – 20 – 50 µM: yellow – green – red – blue, dasatinib 100 nM: magenta.

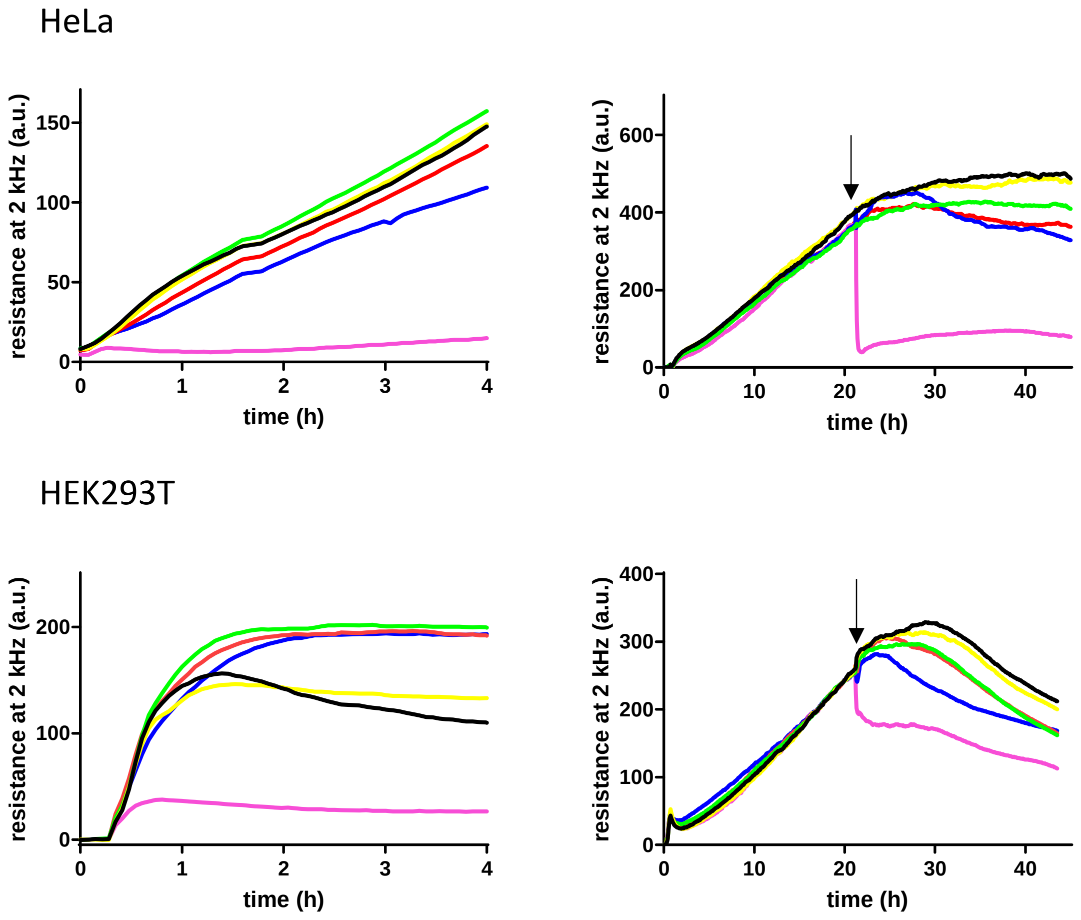

Figure S15: **Effect of JMJD6 silencing by siRNA on PAK band pattern.**
HEK293T were transfected with siRNA JMJD6 and incubated for 48 or 72 h. JMJD6 and PAK expression was then analyzed by western-blotting. Representative examples from repeated experiments.

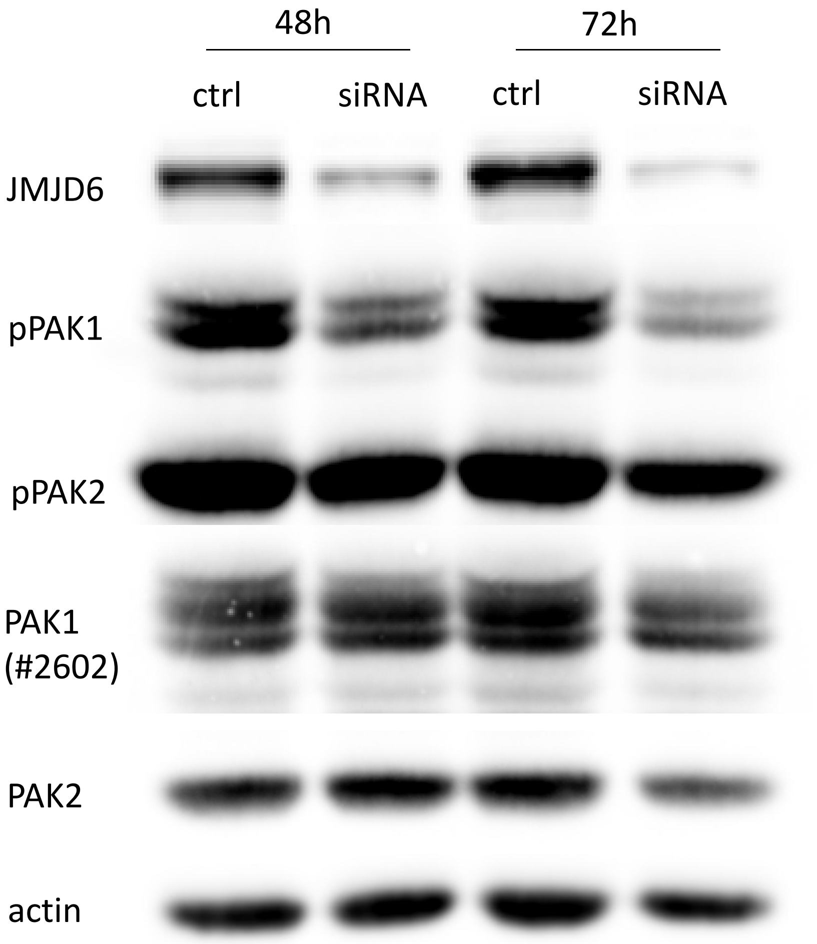

Fig. S16 Full size images of the gels shown in the article figures

Figure 1 Antibody characterization

(a)

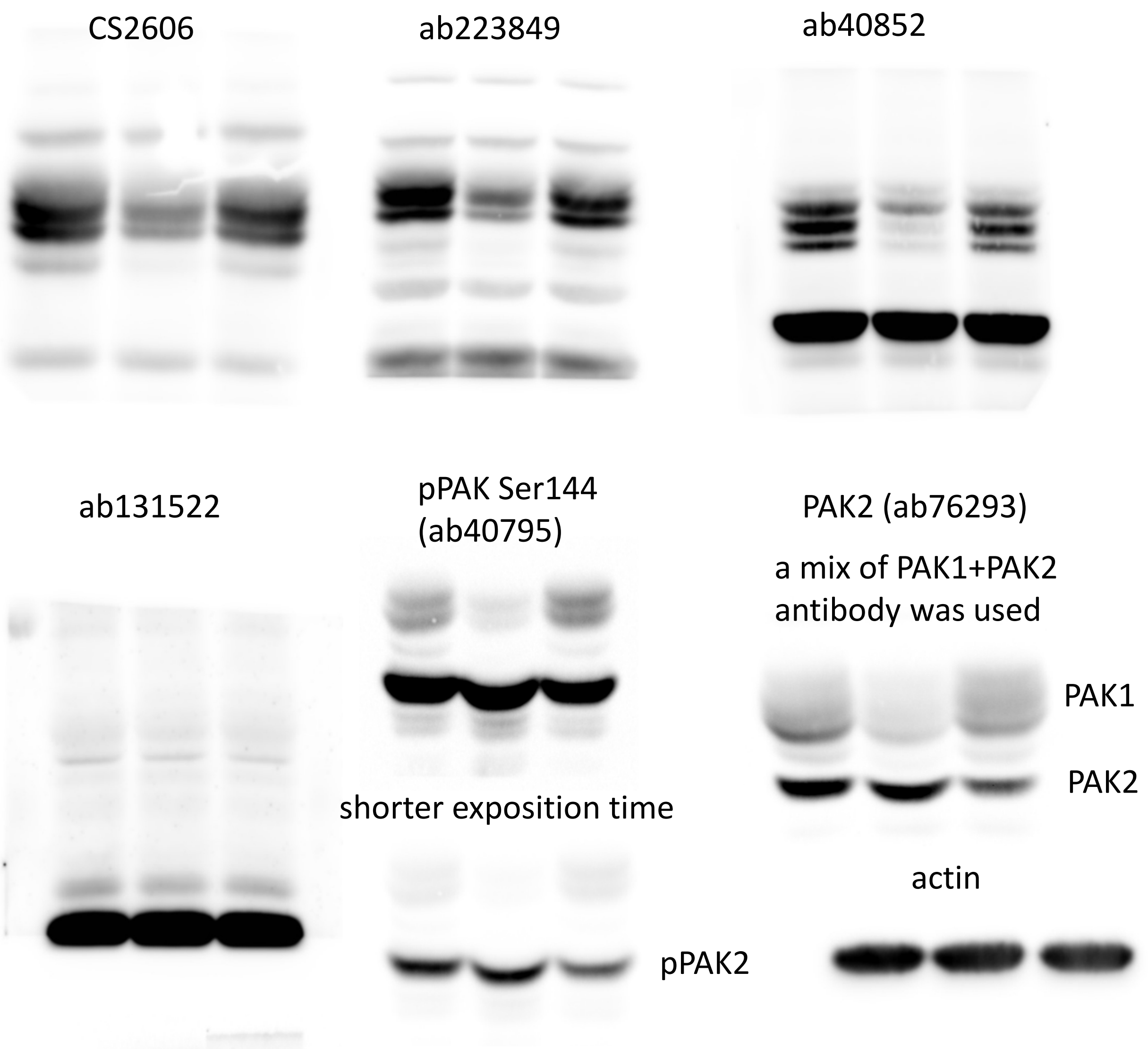

(b)

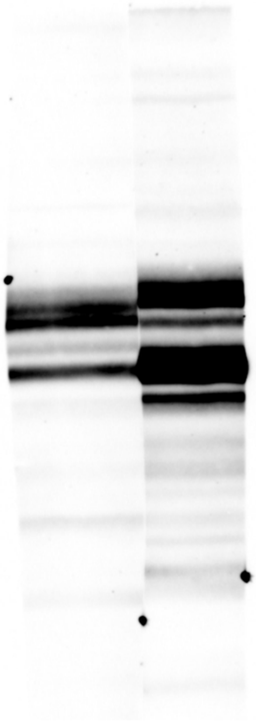

Fig. S16 Full size images of the gels shown in the article figures

Figure 3 Band position for exogenous PAK1 isoforms

CS2606

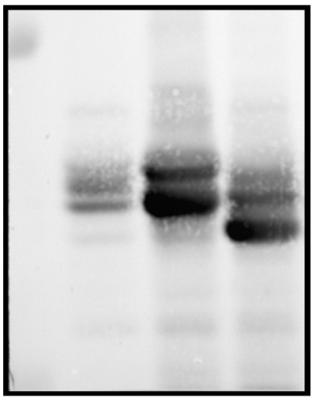

ab223849

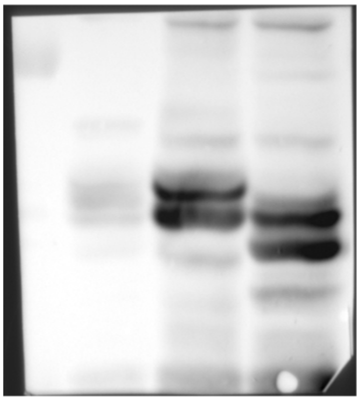

ab40852

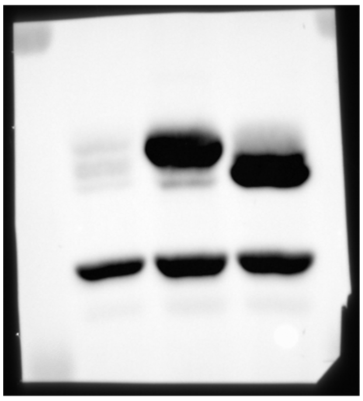

ab131522

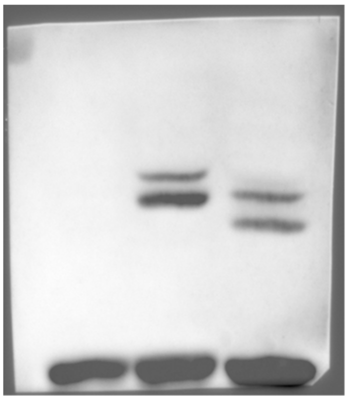

ab40795

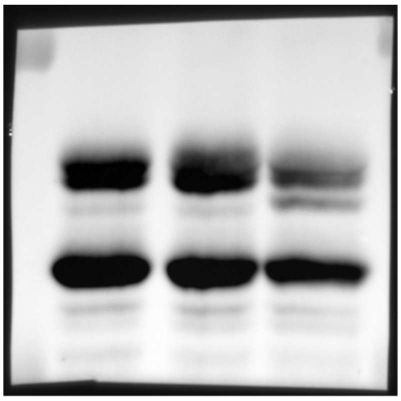

ab75599

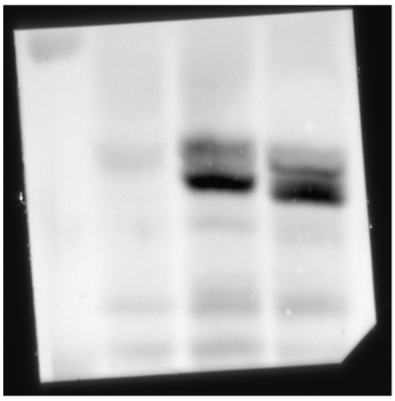

ab76293

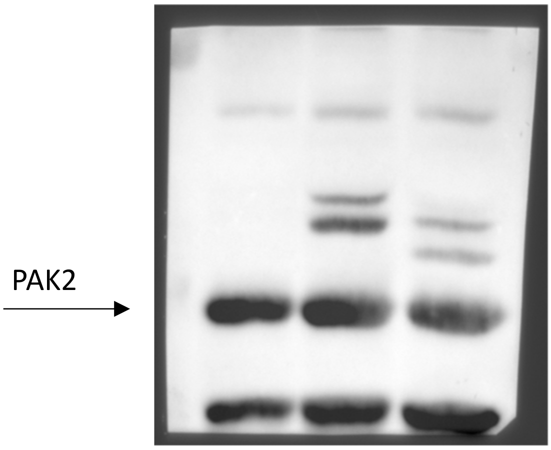

actin

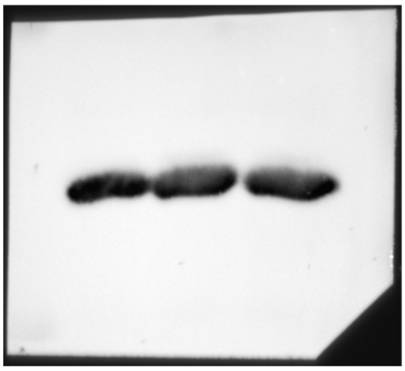

The membrane used for PAK1 detection by ab131522 was reprobed with ab76293 to detect PAK2

Fig. S16 Full size images of the gels shown in the article figures

Figure 4 Effect of alkaline phosphatase

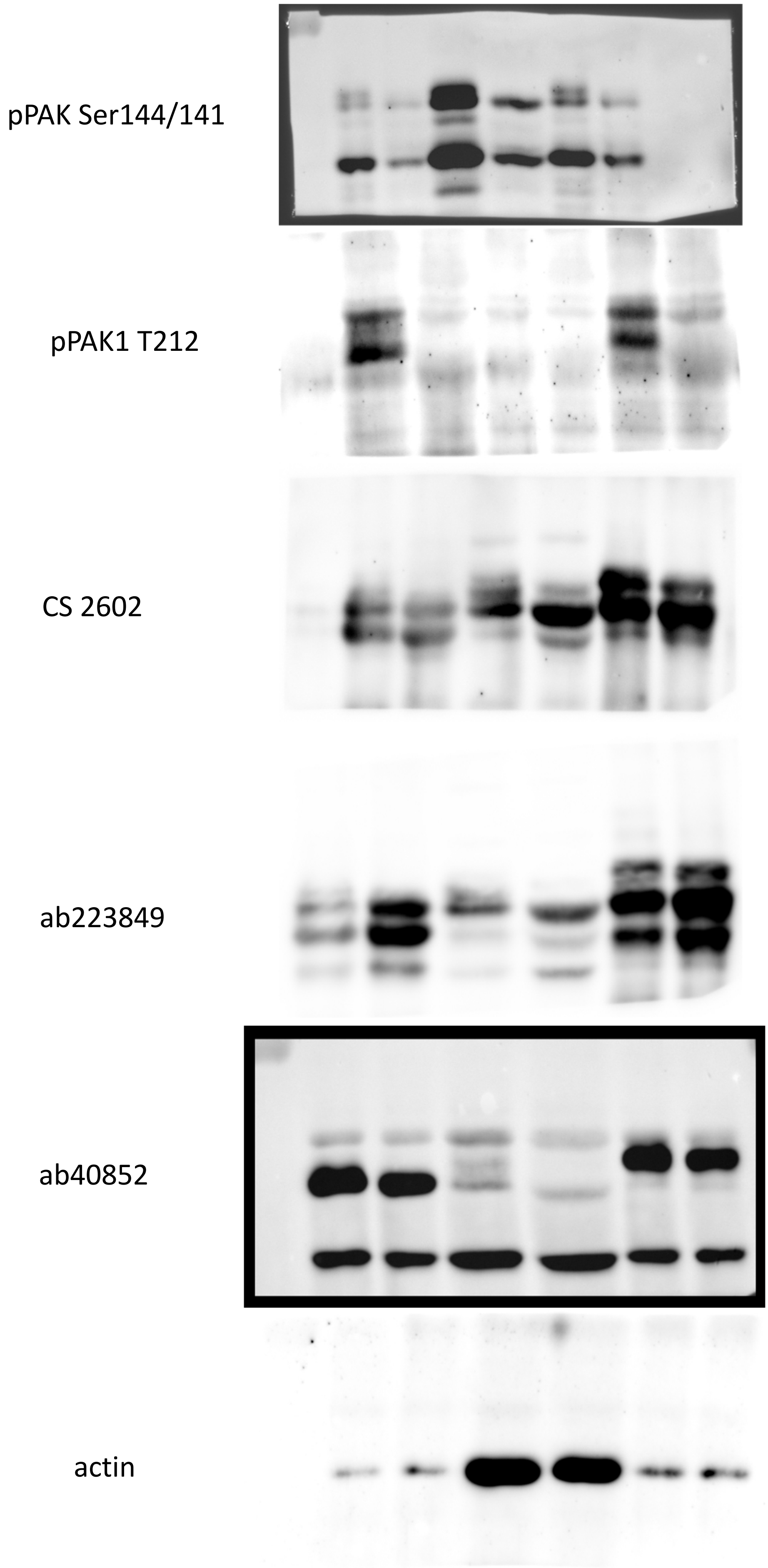

Fig. S16 Full size images of the gels shown in the article figures

Figure 5a Immunoprecipitation experiments

(a) 1: PAK1-full-G + PAK1-full-R, 2: PAK2-G + PAK1-full-R, 3: PAK2-G + mCherry

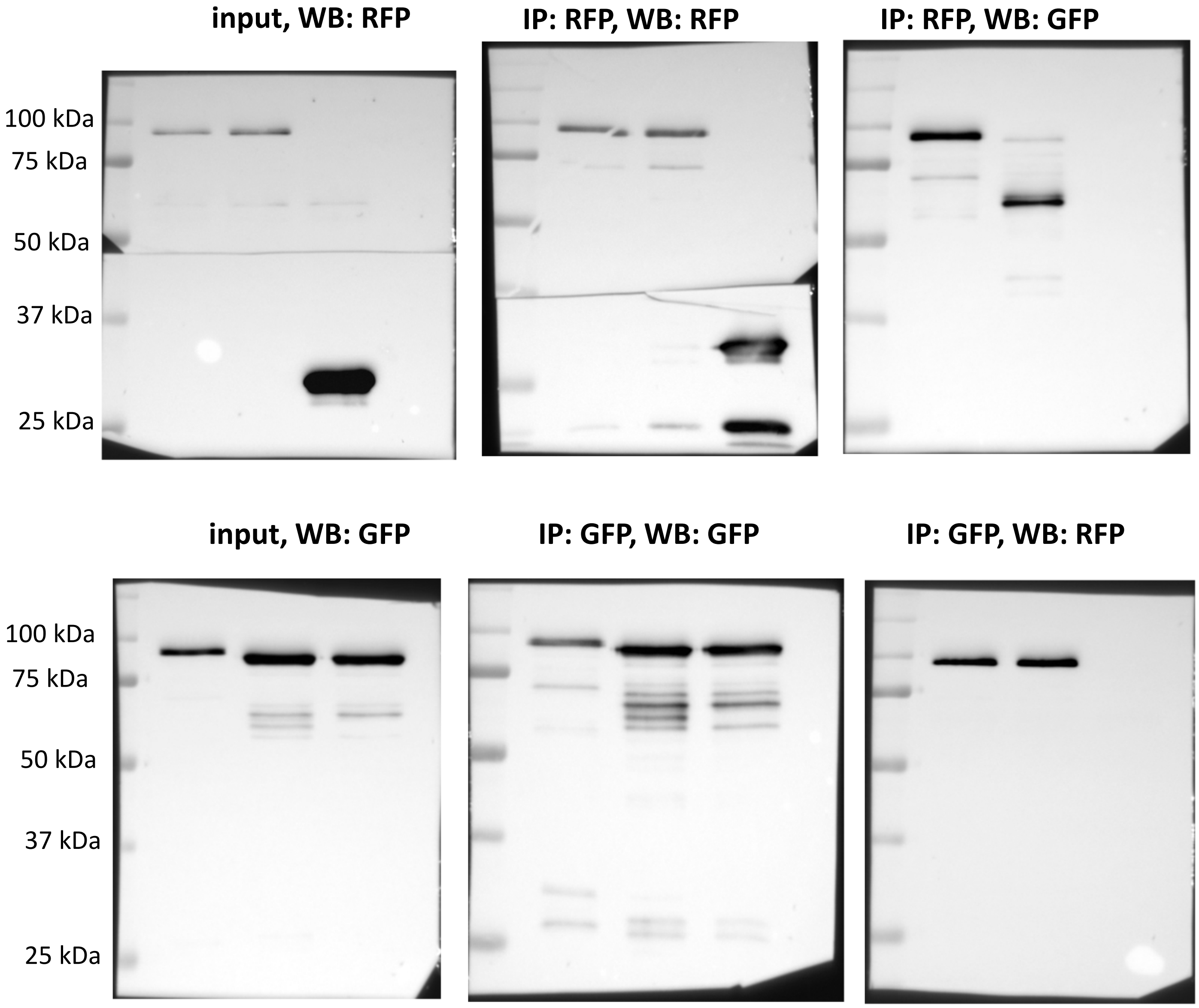

Fig. S16 Full size images of the gels shown in the article figures

Figure 5b Immunoprecipitation experiments

(b) 1: PAKΔ15-G + PAK1-full-R, 2: PAK1-full-G + PAK1-full-R, 3: eGFP + PAK1-full-R

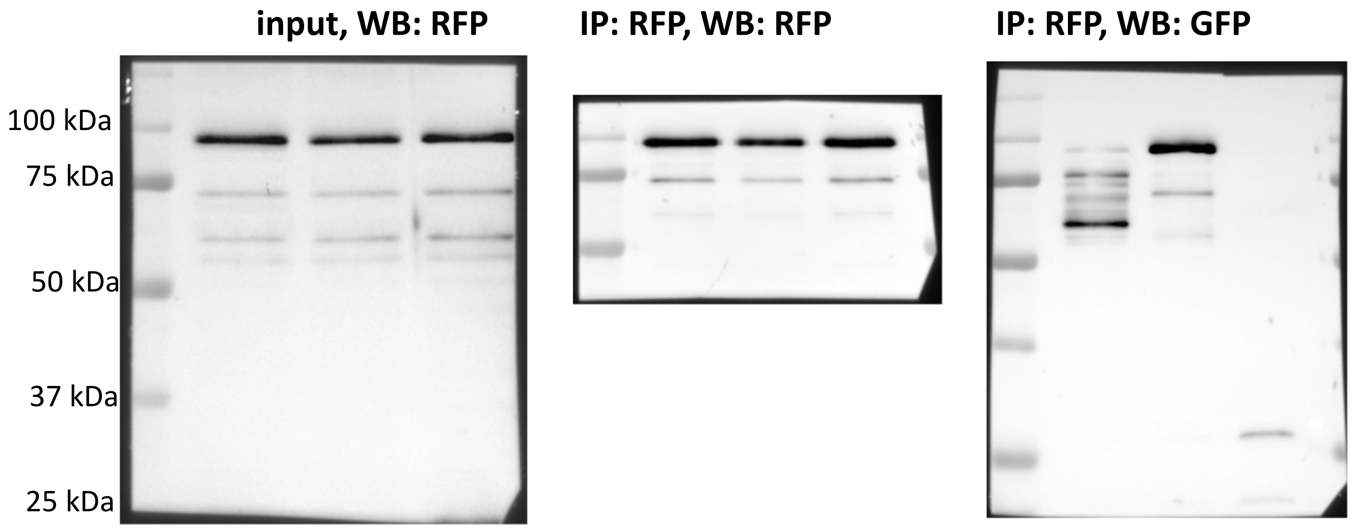

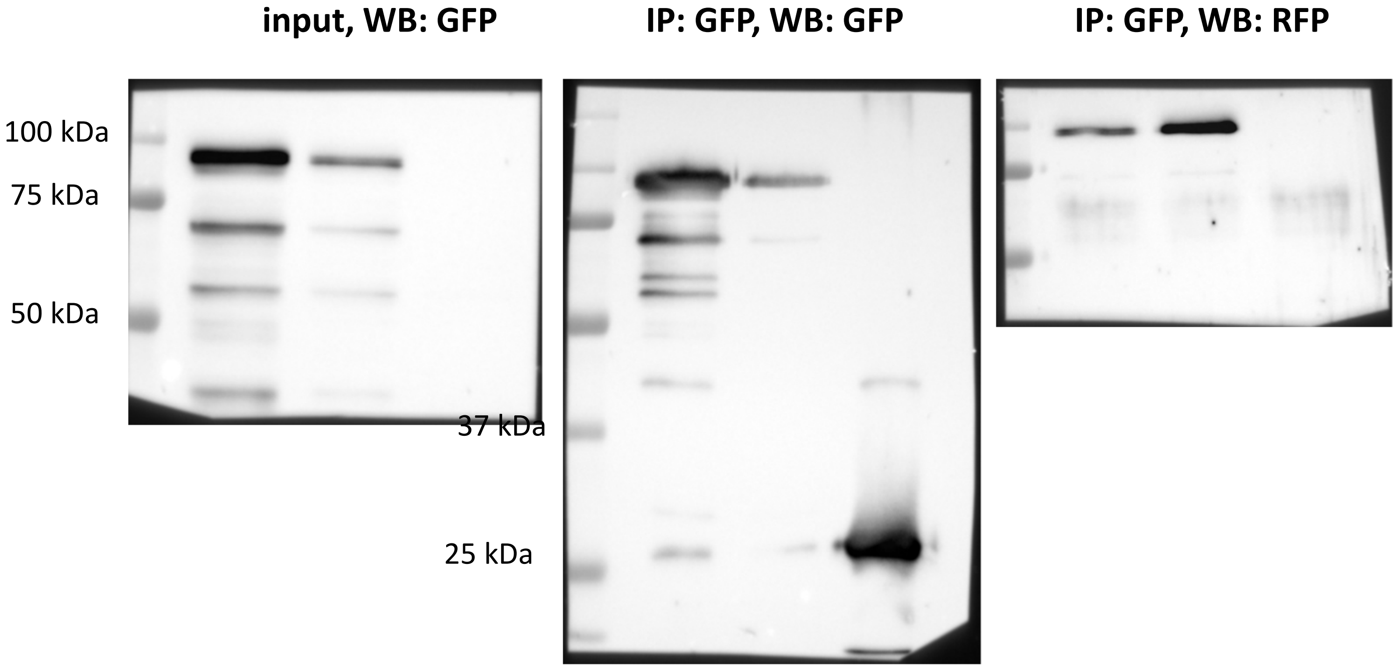

Fig. S16 Full size images of the gels shown in the article figures

Figure 5c Immunoprecipitation experiments

(c) 1: PAK2-G + PAK2-R, 2: PAK1Δ15-G + PAK2-R, 3: eGFP + PAK2-R

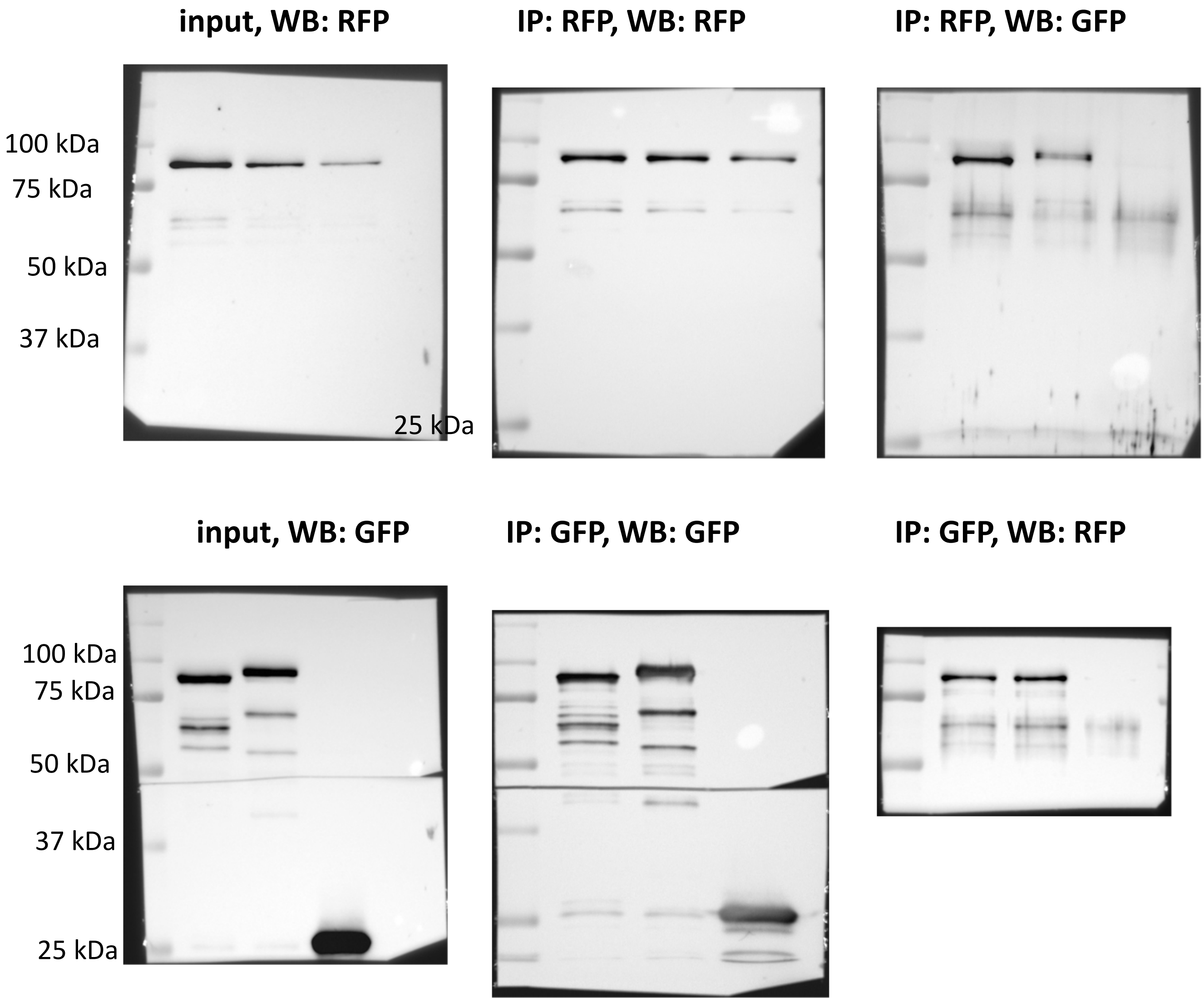

Fig. S16 Full size images of the gels shown in the article figures

Figure 6

Different composition of the sample buffer was tested for gentle denaturation of the native samples. Representative lanes were chosen for Fig. 6 (as indicated by arrows).

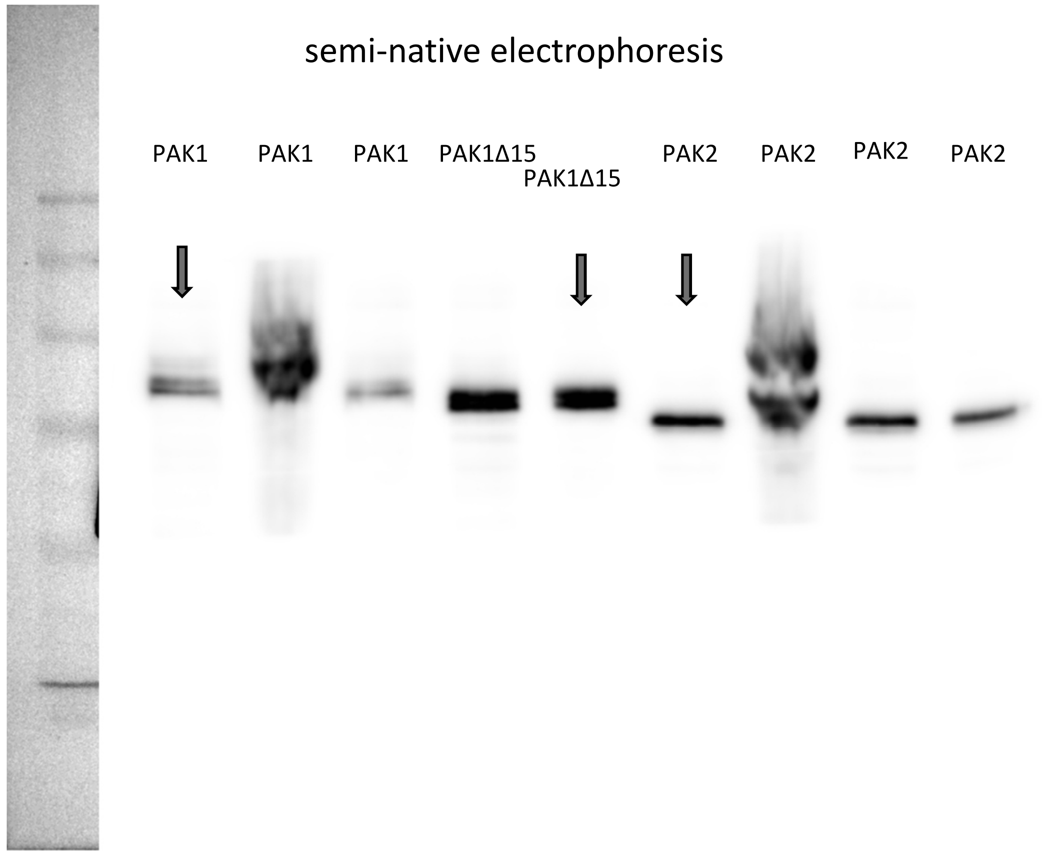

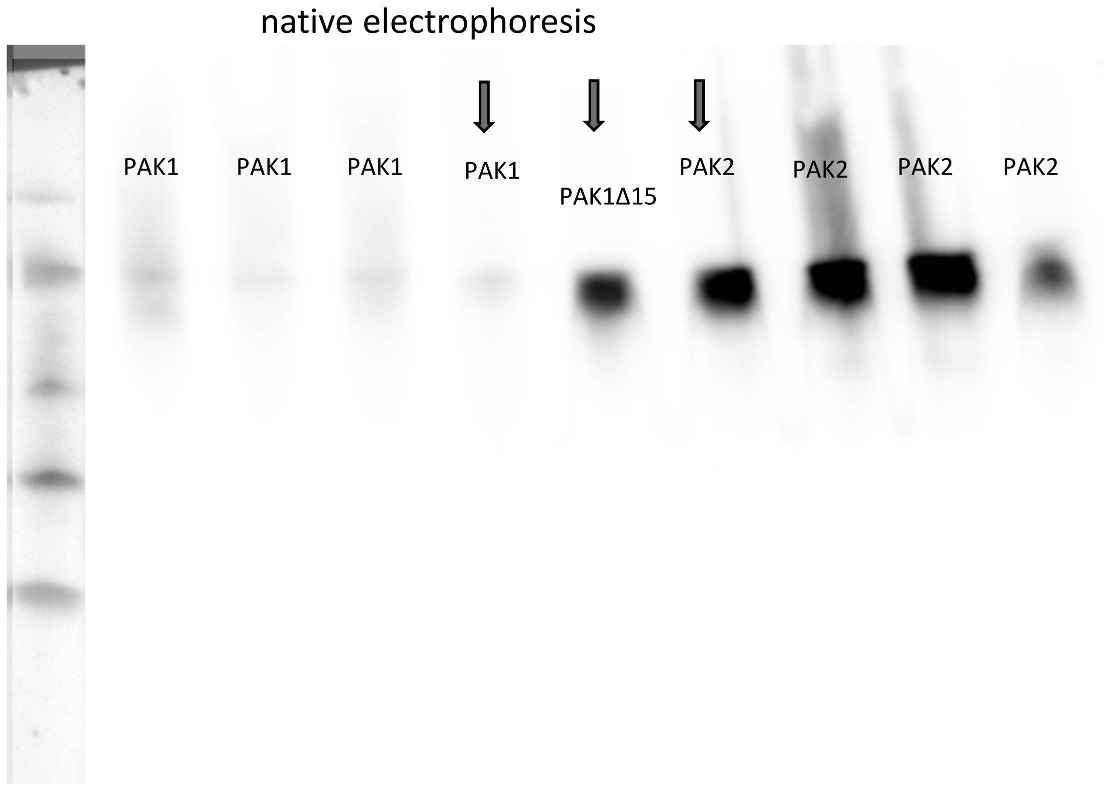

Fig. S16 Full size images of the gels shown in the article figures

Figure 9 Examples of Ser144 dephosphorylation after IPA-3 treatment

pPAK Ser144 (ab40795)

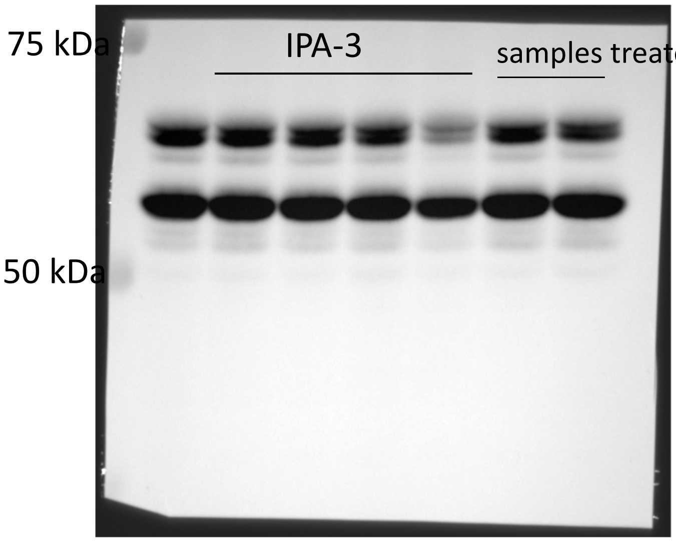

Shorter exposition time on the same membrane was used to quantify pPAK2

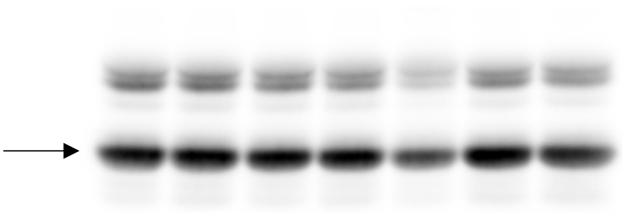

The following membrane was incubated with a mix of PAK1 and PAK2 antibody

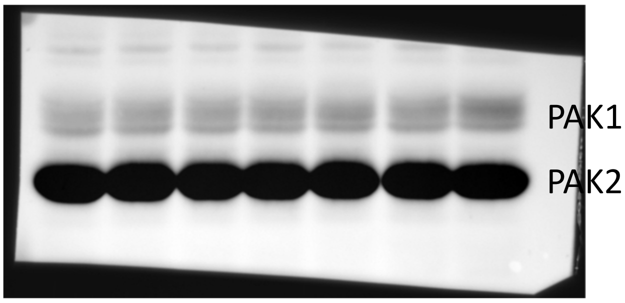

Shorter exposition time with the same membrane was used to quantify PAK2

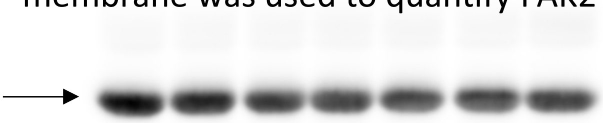

actin

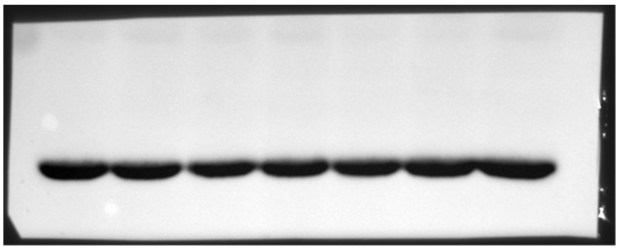

Fig. S16 Full size images of the gels shown in the article figures

Figure 9 Examples of Ser144 dephosphorylation after FRAX597 treatment

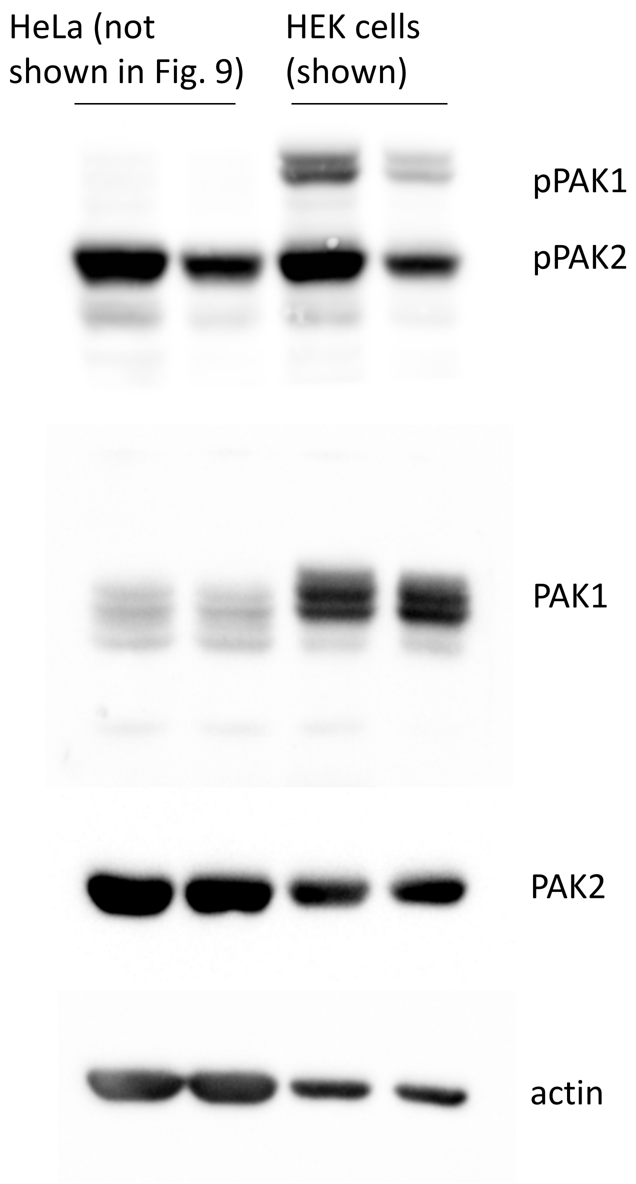

Fig. S16 Full size images of the gels shown in the article figures

Figure 11 controls to siRNA

(a) control to siRNA PAK1 and PAK2

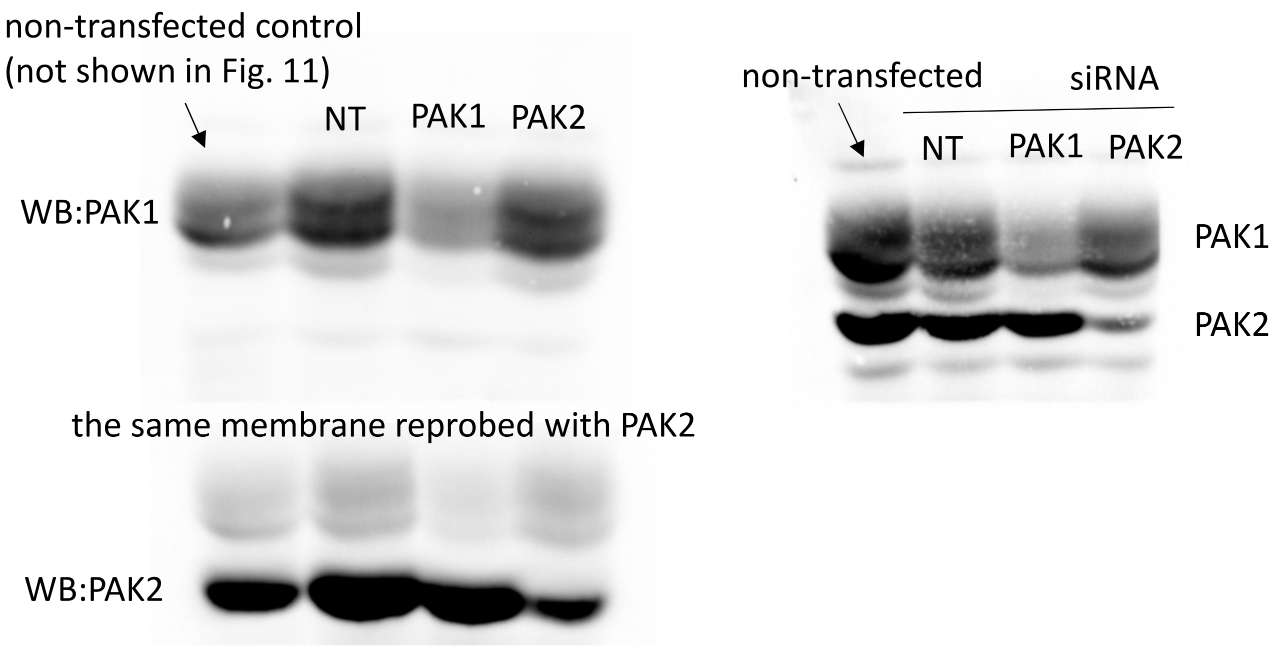

(b) control to siRNA JMJD6

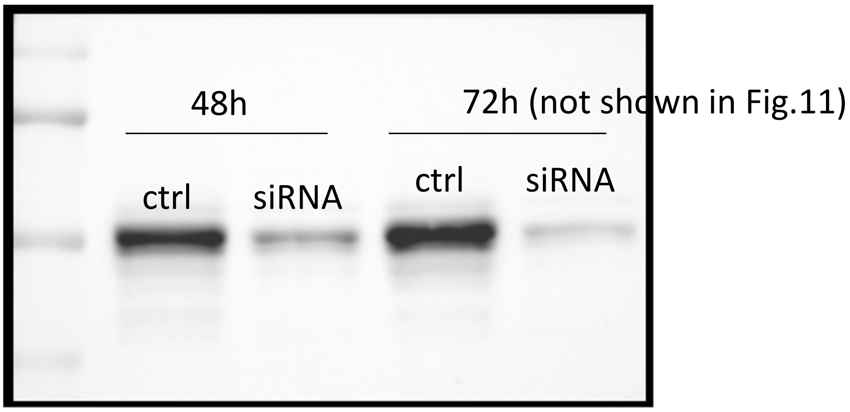

Fig. S16 Full size images of the gels shown in the article figures

Figure S1:**Comparison of signals from different PAK antibodies**

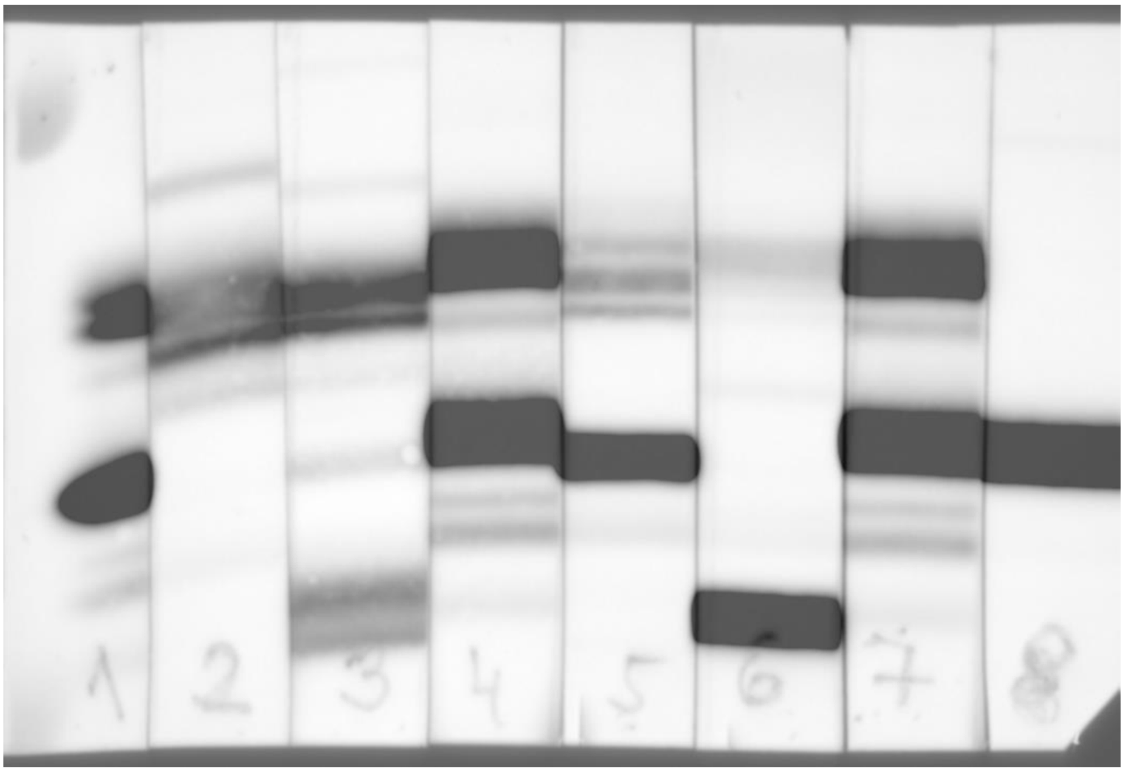

Fig. S16 Full size images of the gels shown in the article figures

Figure S4: **Detection of the truncated PAK2-eGFP in immunoprecipitates**

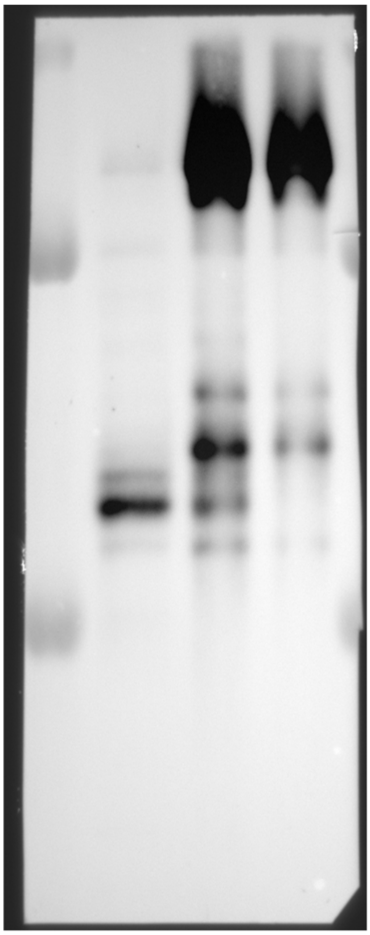

Fig. S16 Full size images of the gels shown in the article figures

Figure S5a: **Co-immunoprecipitation with inverse labeling**

input, WB: RFP

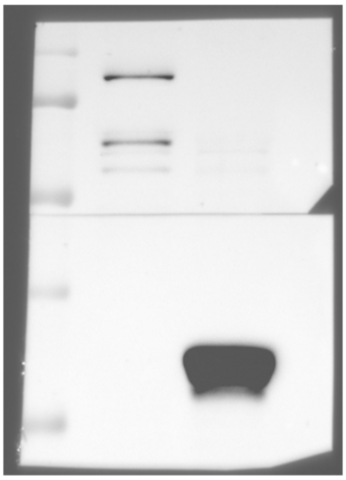

IP: RFP, WB: RFP

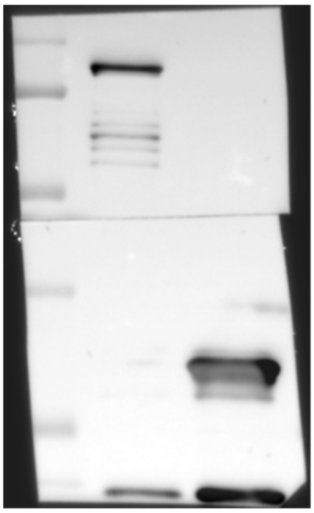

IP: RFP, WB: GFP

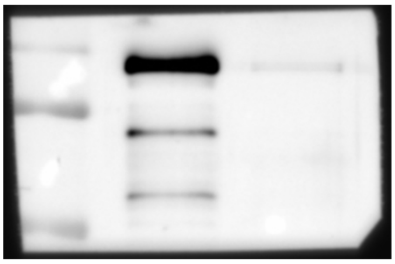

input, WB: GFP

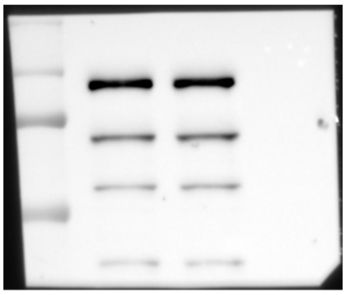

IP: GFP, WB: GFP

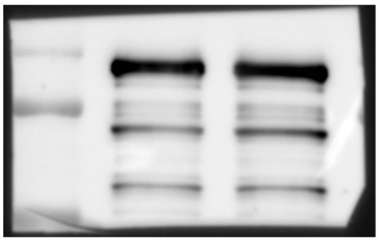

IP: GFP, WB: RFP

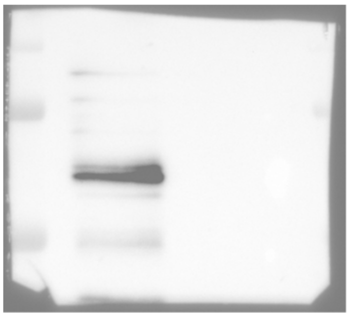

Fig. S16 Full size images of the gels shown in the article figures

Figure S5b: **Effect of caspase inhibition on PAK2-eGFP truncation**

IP: GFP, WB: GFP

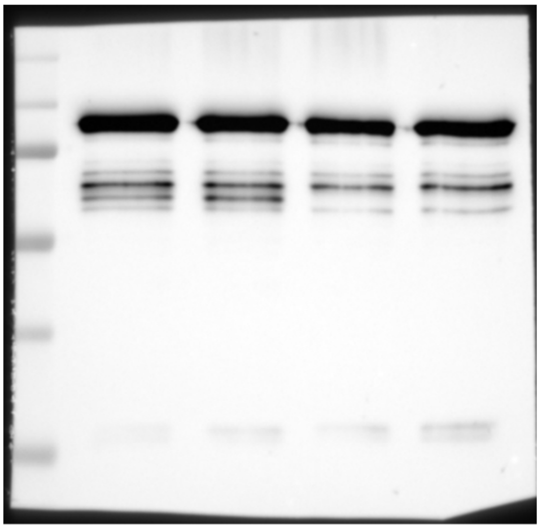

IP: RFP, WB: GFP

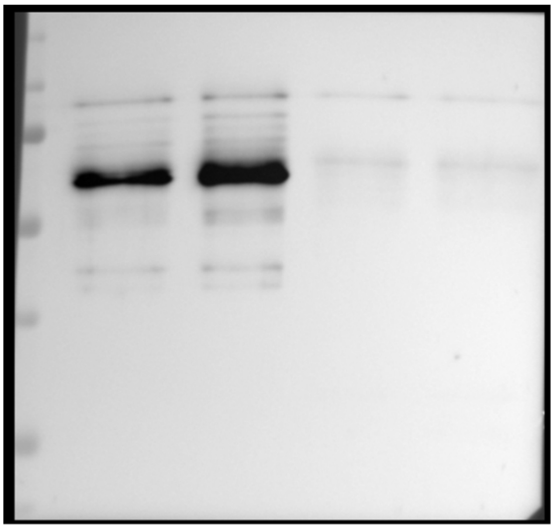

input, WB: PARP

input used for GFP IP

input used for RFP IP (not shown in Fig. S5b)

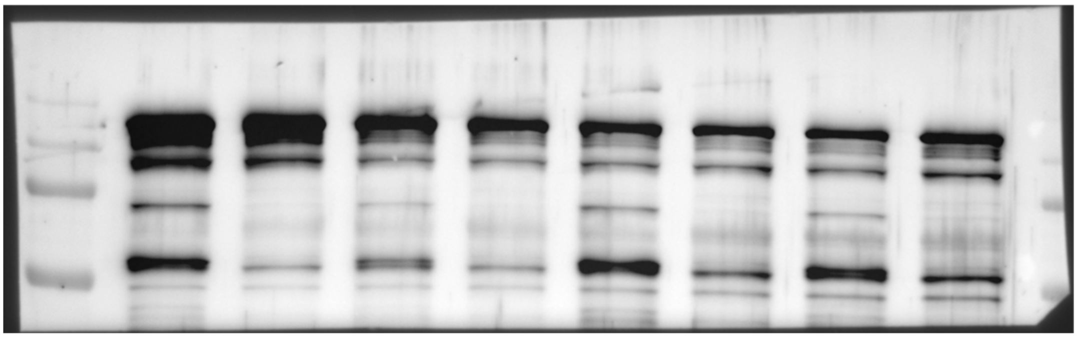

Fig. S16 Full size images of the gels shown in the article figures

Figure S6: (a) **Detection of the endogenous PAK1 in immunoprecipitates.**

anti-GFP

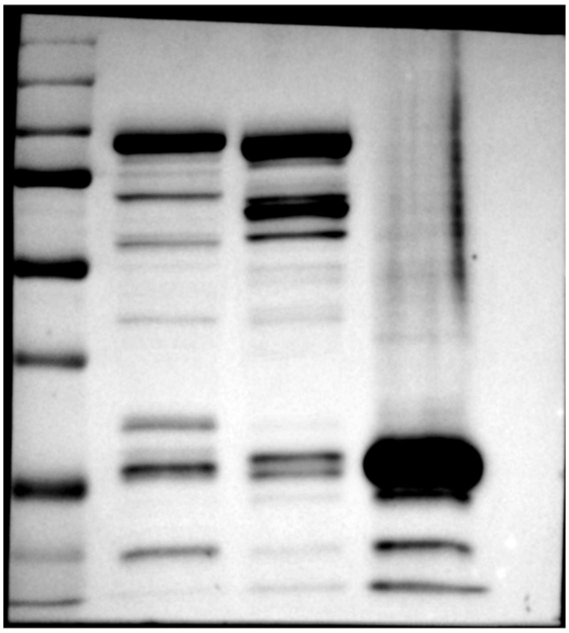

anti-PAK1

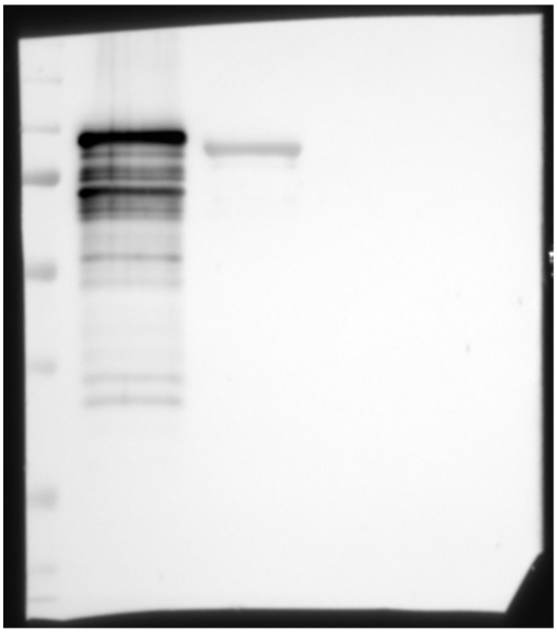

Only the first lanes containing the sample with PAK1-GFP from both membranes are shown in Fig. S6.

(b) **Ser144/141 PAK phosphorylation in cells transfected with PAK-eGFP.**

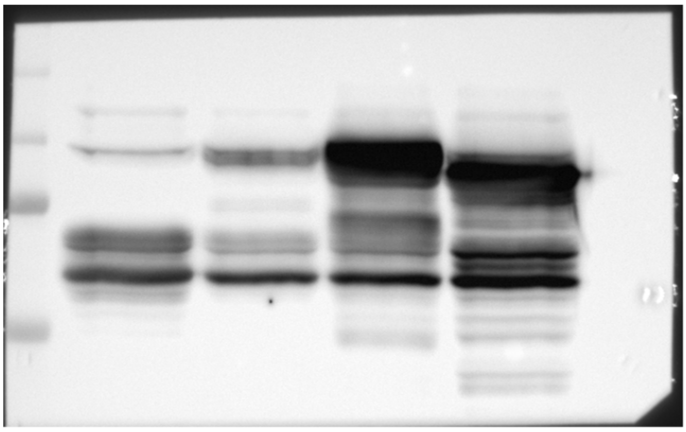

Fig. S16 Full size images of the gels shown in the article figures

Figure S8 **Comparison of PAK1 and PAK2 expression level in HeLa and HEK293T cells.**

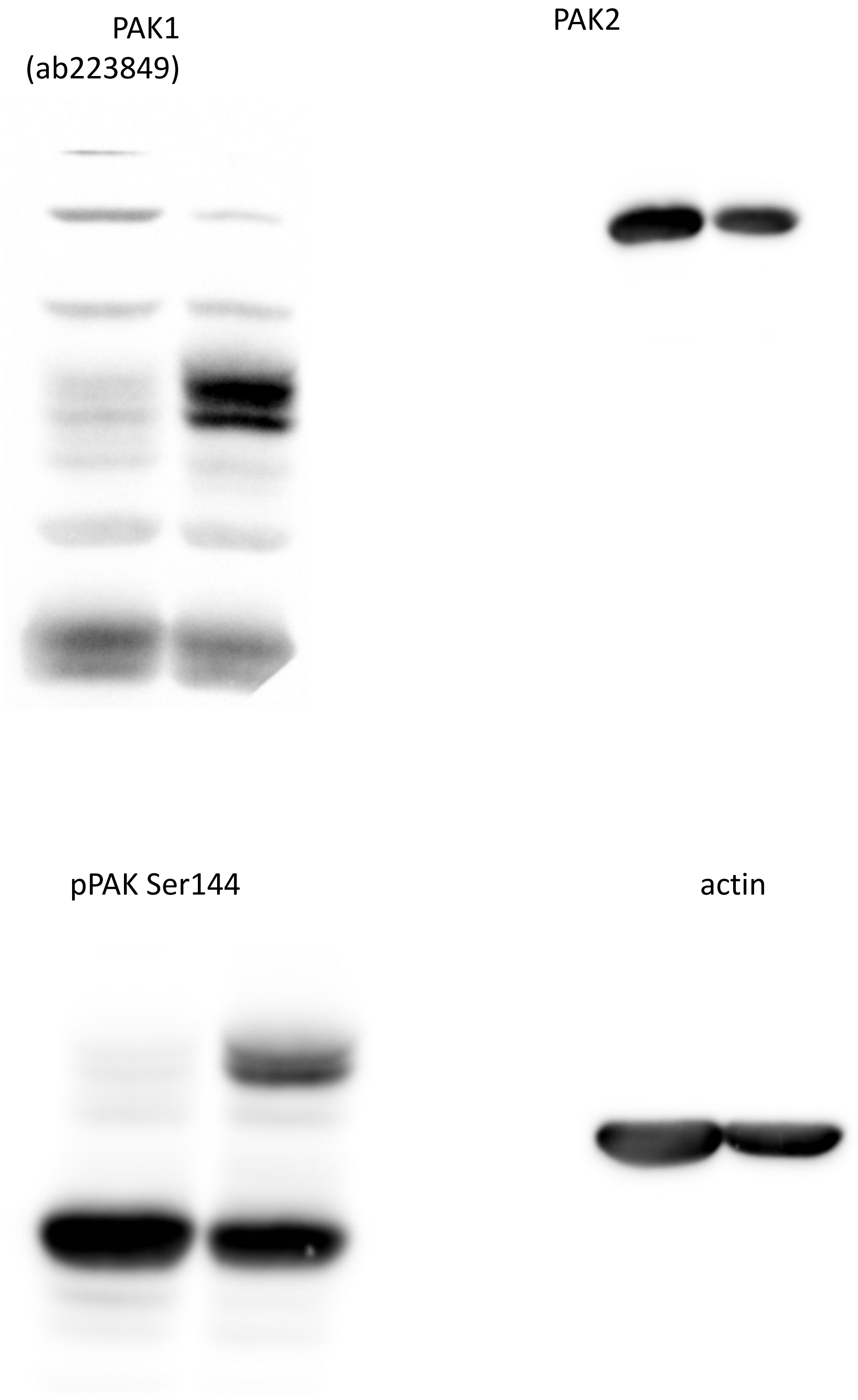

Fig. S16 Full size images of the gels shown in the article figures

Figure S12: **IPA-3-induced changes in T212 and Ser20 PAK1 phosphorylation**

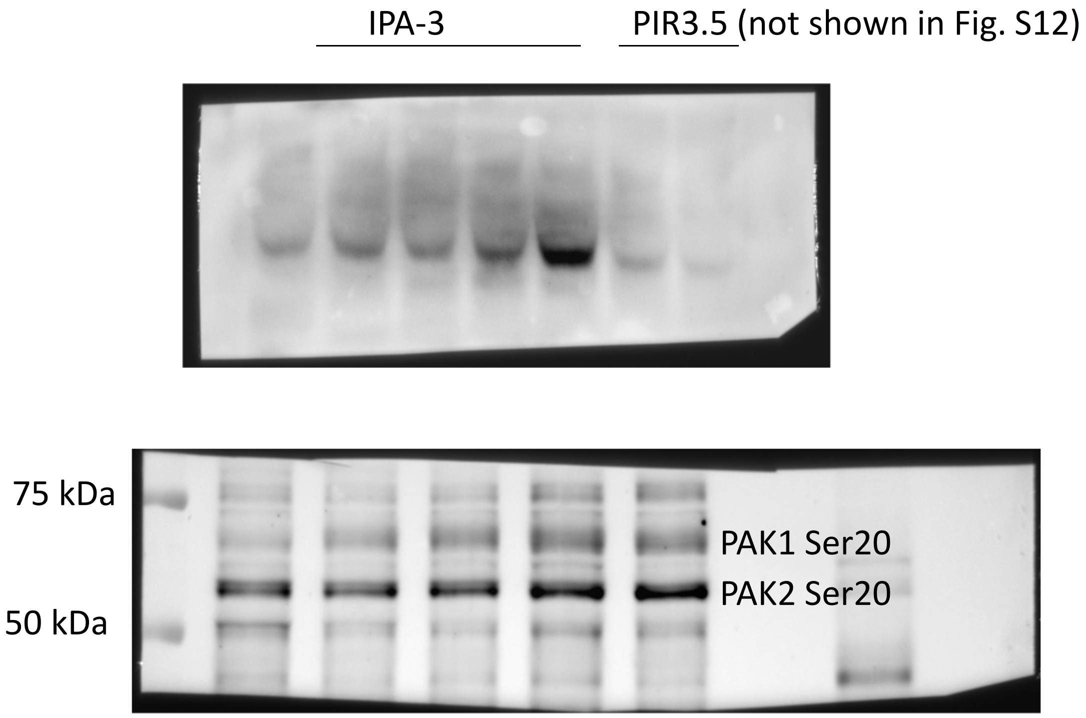

Fig. S16 Full size images of the gels shown in the article figures

Figure S15: **Effect of JMJD6 silencing by siRNA on PAK band pattern.**

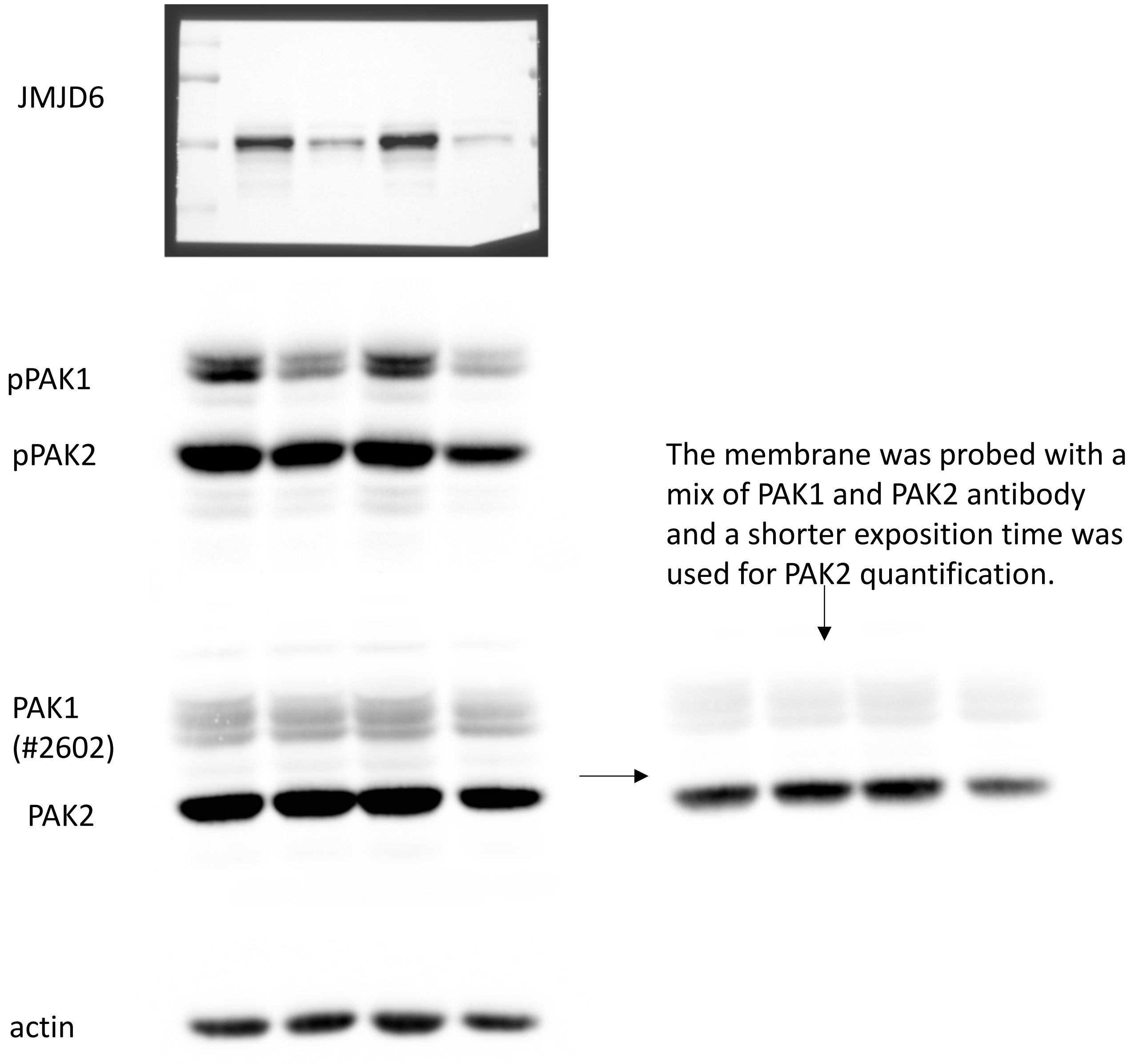

Figure S17: **Comparison of resistance and capacitance signals from ECIS measurement.**
Example of ECIS records from one control well (black) and one IPA-3-treated well (red). The resistance component of the signal measured at 2 kHz (top) is compared with the capacitance component at 64 kHz (bottom). The response to IPA-3 addition (marked by the arrow) is larger in the 2 kHz resistance signal.

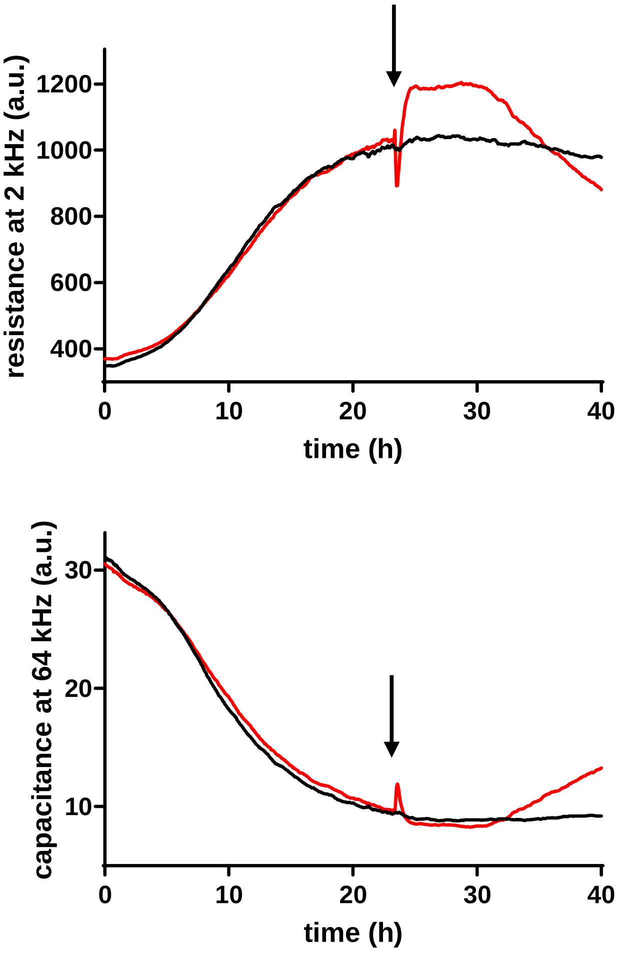

Supplement: Supplementary file 1 — Supplementary Information [file 41598_2019_53665_MOESM1_ESM.pdf]
